# Zebrafish reporter lines reveal *in vivo* signaling pathway activities involved in pancreatic cancer

**DOI:** 10.1242/dmm.014969

**Published:** 2014-05-30

**Authors:** Marco Schiavone, Elena Rampazzo, Alessandro Casari, Giusy Battilana, Luca Persano, Enrico Moro, Shu Liu, Steve D. Leach, Natascia Tiso, Francesco Argenton

**Affiliations:** 1 Department of Biology, University of Padua, 35131 Padua, Italy.; 2 Department of Molecular Medicine, University of Padua, 35131 Padua, Italy.; 3 Department of Woman and Child Health, University of Padua, 35131 Padua, Italy.; 4 Department of Surgery and The McKusick-Nathans Institute of Genetic Medicine Johns Hopkins University School of Medicine, Baltimore, MD 21205, USA.

**Keywords:** Zebrafish, Pancreatic adenocarcinoma, Medulloblastoma, KRAS, Reporters, TGFβ, Notch, Shh

## Abstract

Pancreatic adenocarcinoma, one of the worst malignancies of the exocrine pancreas, is a solid tumor with increasing incidence and mortality in industrialized countries. This condition is usually driven by oncogenic *KRAS* point mutations and evolves into a highly aggressive metastatic carcinoma due to secondary gene mutations and unbalanced expression of genes involved in the specific signaling pathways. To examine *in vivo* the effects of KRAS^G12D^ during pancreatic cancer progression and time correlation with cancer signaling pathway activities, we have generated a zebrafish model of pancreatic adenocarcinoma in which eGFP-KRAS^G12D^ expression was specifically driven to the pancreatic tissue by using the GAL4/UAS conditional expression system. Outcrossing the inducible oncogenic KRAS^G12D^ line with transgenic zebrafish reporters, harboring specific signaling responsive elements of transcriptional effectors, we were able to follow TGFβ, Notch, Bmp and Shh activities during tumor development. Zebrafish transgenic lines expressing eGFP-KRAS^G12D^ showed normal exocrine pancreas development until 3 weeks post fertilization (wpf). From 4 to 24 wpf we observed several degrees of acinar lesions, characterized by an increase in mesenchymal cells and mixed acinar/ductal features, followed by progressive bowel and liver infiltrations and, finally, highly aggressive carcinoma. Moreover, live imaging analysis of the exocrine pancreatic tissue revealed an increasing number of KRAS-positive cells and progressive activation of TGFβ and Notch pathways. Increase in TGFβ, following KRAS^G12D^ activation, was confirmed in a concomitant model of medulloblastoma (MDB). Notch and Shh signaling activities during tumor onset were different between MDB and pancreatic adenocarcinoma, indicating a tissue-specific regulation of cell signaling pathways. Moreover, our results show that a living model of pancreatic adenocarcinoma joined with cell signaling reporters is a suitable tool for describing *in vivo* the signaling cascades and molecular mechanisms involved in tumor development and a potential platform to screen for novel oncostatic drugs.

## INTRODUCTION

Pancreatic adenocarcinoma is one of the most aggressive cancers in industrial countries, and its incidence and mortality is still increasing. The poor survival rate of this malignant disease indicates that current interventions to prevent, diagnose and cure pancreatic adenocarcinoma are far from satisfactory. Identification of the molecular and biochemical processes that regulate the onset and progression of pancreatic adenocarcinoma is of great relevance for therapeutic purposes. In general, crucial signaling transduction pathways involved in cell proliferation, stem-cell maintenance and differentiation during embryonic development appear disrupted during tumor formation. In pancreatic adenocarcinoma of both acinar and ductal origins most pathways, including Sonic Hedgehog (Shh), Wnt, Notch and transforming growth factor β (TGFβ) signaling, have been shown to be dysregulated (Bailey and Leach, 2012). During embryonic development, Shh plays a major role in stem-cell proliferation (Wu et al., 2010); the Wnt pathway is involved in cell proliferation and differentiation (Yang, 2012); Notch is responsible for stem-cell maintenance (Kwon et al., 2012); and TGFβ controls cell and tissue homeostasis, favoring cell apoptosis by cross-talking with other pathways such as p53 (Elston and Inman, 2012). TGFβ is also involved in tissue morphogenesis, in cooperation with Wnt pathway, by controlling epithelial-to-mesenchymal transition (EMT) and cell migration (Jing et al., 2011; Zhou et al., 2012). During carcinogenesis, Shh and Notch pathways seem to be involved in tumor onset, together with genomic instability, whereas Wnt and TGFβ appear activated in cancer progression by eliciting cell migration or neo-angiogenesis through reciprocal cross-talk or by interactions with other pathways (McCleary-Wheeler et al., 2012). For instance, non-canonical Wnt has been proposed to be involved in cell proliferation and metastasis by cross-talking with TGFβ (McCleary-Wheeler et al., 2012). Several *in vitro* and *in vivo* studies demonstrated that, within the tumor cell compartment, TGFβ has a dual role. By inhibiting cell growth, it has a tumor suppressor function at early tumor stages, whereas at later stages it mediates oncogenic effects (Heldin and Moustakas, 2012; McCleary-Wheeler et al., 2012; Truty and Urrutia, 2007).

Animal models of human cancers provide unique insights into the study and understanding of molecular pathways involved at both early and late stages of malignant diseases, easing the discovery of biomarkers and specific targets for new or more effective drug therapies (Etchin et al., 2011; Herreros-Villanueva et al., 2012a; Herreros-Villanueva et al., 2012b; Stoletov and Klemke, 2008). We focused on the vertebrate teleost *Danio rerio* to uncover *in vivo* the complex pathways regulating the biological processes underlying pancreatic adenocarcinoma (Bailey and Leach, 2012; Goldsmith and Jobin, 2012). Pancreatic adenocarcinoma is a solid tumor originated by the epithelial cells of pancreatic duct and by changes in the exocrine acinar structure as it reverts into ductal structure (Bardeesy and DePinho, 2002; Esposito et al., 2007). Many studies have focused on point mutations causing the constitutive activation of the oncogene *KRAS* which, in turn, activates mechanisms bringing pancreatic cancer onset (Park et al., 2008). Point mutations of the amino acid residue at position 12 of KRAS protein (G12V and G12D) are the most frequent changes found during the first stages of pancreatic adenocarcinoma. Subsequent unbalanced expression of several genes such as *P53*, *STAT3* (Corcoran et al., 2011), *SMAD4* and *ARF/INK4* (Aguirre et al., 2003) brings carcinoma *in situ* and, further, to metastatic carcinoma (Bardeesy et al., 2006). Several studies on zebrafish and mice showed the involvement of Shh signaling during early stages and its role in the stimulation of TGFβ1 activity in duct cells during pancreatic fibrosis and at later stages of pancreatic ductal adenocarcinoma (Jung et al., 2011). However, how the unbalance of Shh, TGFβ, Notch and Bmp signaling pathways reflects in pancreatic adenocarcinoma progression remains to be unveiled. To trace *in vivo* the activity of Shh, TGFβ, Notch and Bmp, we used zebrafish transgenic lines expressing the fluorescent reporter mCherry under control of specific responsive elements recognized by downstream regulators of each pathway.

**TRANSLATIONAL IMPACT**

**Clinical issue**

With an overall 5-year survival rate of only 3–5% after diagnosis, pancreatic adenocarcinoma is one of the most aggressive malignancies in the industrialized world. Surgery and traditional combined therapies are not effective enough to eradicate this deadly disease. Understanding the cell origin and molecular mechanisms involved in its onset and progression is a major step towards the development of novel drugs against pancreatic adenocarcinoma, and many rodent models have been developed during the last ten years. Zebrafish can provide a useful complementary model to explore the characteristics of the disease *in vivo* because of the organism’s amenability to live imaging and the conserved genetic control for pancreatic development between fish and mammals. The aim of this study was to use zebrafish to explore *in vivo* the tumorigenic action of a constitutively active mutant version of the oncogene *KRAS*, implicated in several types of pancreatic adenocarcinoma.

**Results**

Using promoter and enhancer elements of *ptf1a*, a transcription factor expressed in the exocrine pancreas and in cerebellar GABAergic neurons, KRAS^G12D^ was specifically expressed in the pancreas and, concomitantly, in the cerebellum using a Gal4/UAS inducible system. By using mCherry cell signaling reporter lines, the authors observed dysregulated activity of the Notch pathway during the early stages and dysregulation of Smad3/TGFβ and Shh pathways during the later stages of pancreatic adenocarcinoma. Upon comparison of pancreatic adenocarcinoma with concomitant *ptf1a*-induced medulloblastoma in the cerebellum, they conclude that TGFβ, Shh and Notch are involved in both cancers at different stages of carcinogenesis. Furthermore, they provide evidence that Smad3/TGFβ is controlled by KRAS in both cancers. Interestingly, Notch and Shh signaling activities differed during tumor onset in medulloblastoma compared with pancreatic adenocarcinoma, indicating tissue-specific regulation of these cell signaling pathways.

**Implications and future directions**

This study provides insight into the signaling pathways involved in pancreatic adenocarcinoma onset and progression. The identification of mechanisms regulating the hallmarks of this aggressive disease and of candidate molecules (Notch, TGFβ and Shh ligands and their effectors) with the potential to interfere with cancer progression is an important step towards new therapies for pancreatic adenocarcinoma. Furthermore, the study provides a new animal model of the disease, which, coupled with transgenic zebrafish reporter lines, provides a powerful tool for tracing the *in vivo* dynamics of pancreatic tumorigenesis and for screening candidate drugs. In the long-term, stratification of the many types of pancreatic cancer according to genotype and developmental stage could pave the way to the development of molecularly targeted therapies.

To obtain a zebrafish model for pancreatic carcinogenesis, we took inspiration from a zebrafish model established by Park et al. (Park et al., 2008) based on the constitutive activation of *KRAS^G12V^* oncogene in the pancreas under control of ptf1a promoter elements. In this work, a new zebrafish model of pancreatic adenocarcinoma was reproduced by using the conditional Gal4/UAS expression system (Liu and Leach, 2011), driving the expression of mutated KRAS^G12D^ in the exocrine pancreas of zebrafish derived from outcrosses with TGFβ, Notch, Bmp and Shh reporter lines expressing the fluorescent reporter mCherry. The selected *ptf1a* promoter also drives conditional KRAS^G12D^ expression in cerebellum; thus, we were able to assess *KRAS* activity during the early stages of cerebellar development by obtaining a concomitant model of putative pediatric medulloblastoma (MDB) (Gilbertson et al., 2006). By comparing the expression of mCherry reporters we were able to reveal *in vivo* signaling pathways elicited after oncogenic *KRAS* constitutive activation in both pancreas and cerebellum, showing *in vivo* how TGFβ, Notch and Shh are involved in pancreatic adenocarcinoma and during MDB carcinogenesis.

## RESULTS

### Expression of eGFP-KRAS^G12D^ and Kaplan-Meier analysis in a pancreatic adenocarcinoma and medulloblastoma zebrafish model based on a Gal4/UAS expression system

**Fig. 1.**
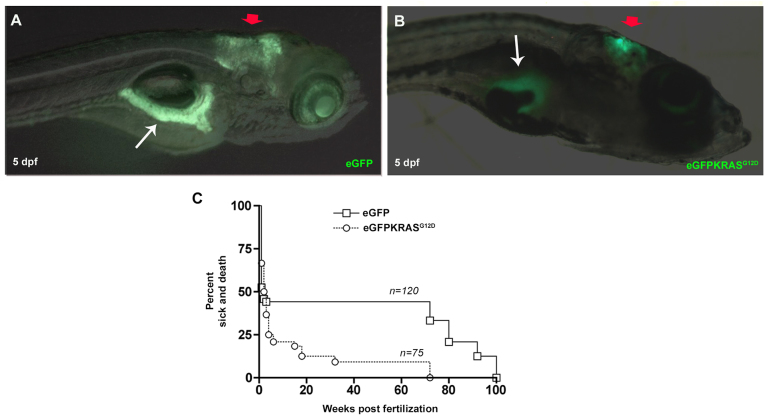
**Tissue specificity expression of eGFP and eGFP-KRAS^G12D^ under control of *ptf1a* promoter and Kaplan–Meier analysis.** (A,B) Lateral views, anterior to the right showing that EGFP (A) and eGFP-KRAS^G12D^ (B) are specifically expressed in cerebellum (red arrows) and pancreas (white arrows) of larvae observed at 5 dpf. (C) Kaplan–Meier analysis of dead or severely sick animals revealed lethality for about 92% of *Tg(ptf1a:Gal4)/UAS:eGFP-KRAS^G12D^* as measured at 24 wpf. Differences between *Tg(ptf1a:eGFP)* and *Tg(ptf1a:Gal4)/UAS:eGFP-KRAS^G12D^* were statistically significant with *P*<0.05. Number of sample for each group are indicated.

To generate a zebrafish model of pancreatic adenocarcinoma, eggs derived from *Tg(ptf1a:Gal4)* outcrosses were injected with *Tol2(UAS:eGFP-KRAS^G12D^)* plasmid (Liu and Leach, 2011; Park et al., 2008); we call these samples “*Tg(ptf1a:Gal4)/UAS:eGFP-KRAS^G12D^* injected”. As a control, we used outcrosses of the stable transgenic line *Tg(ptf1a:eGFP)* expressing cytoplasmic enhanced green fluorescent protein (eGFP) in cells from both pancreas and cerebellum. In particular, we observed tissue-specific expression of eGFP both in pancreas and cerebellum in all 75 *Tg(ptf1a:eGFP)* collected controls, faithfully recapitulating the endogenous pattern of *ptf1a* expression (Fig. 1A) (Lin et al., 2004; Sellick et al., 2004; Zecchin et al., 2004). In order to obtain a significant number of cancer lesions, we collected 120 *Tg(ptf1a:Gal4)/UAS:eGFP-KRAS^G12D^* injected animals. All the selected *Tg(ptf1a:Gal4)/UAS:eGFP-KRAS^G12D^* injected larvae expressed eGFP-KRAS^G12D^ in cerebellum but only 50% of them were also expressing eGFP-KRAS^G12D^ in the pancreas (Fig. 1B). This might reflect the fact that, as already described, expression of *ptf1a* in cerebellum is stronger than in pancreas (Zecchin et al., 2004).

**Fig. 2.**
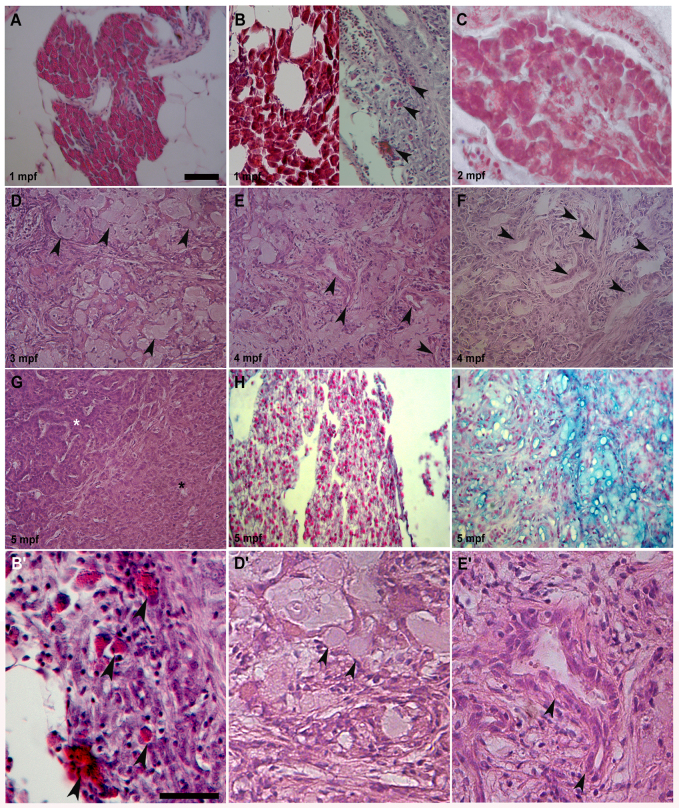
**Different pancreas transformations induced by KRAS^G12D^**. (A) Control exocrine pancreas characterized by organized acinar clusters surrounded by fat cells and ducts. (B, left; C). Pre-tumoral acinar hyperplasia induced by KRAS^G12D^ seen in 6 out of 16 analyzed samples at 1 and 2 mpf. Acinar tissue extension over the entire exocrine pancreas replaced the ductal ephitelium. (B, right) Pre-tumoral fibrotic pancreas tissue with stromal enrichment, reactive ducts and a few acinar cells (arrowheads) interspersed inside disorganized exocrine tissue was seen in 10 out of 16 collected samples. (D) End-stage mucinous pancreatic adenocarcinoma of acinar origin with goblet cells (arrowheads) interspersed into exocrine tissue. (E) End-stage mucinous pancreatic adenocarcinoma with reactive ducts (arrowheads), increasing stroma and goblet cells. (F) End-stage mucinous adenocarcinoma with reactive ducts losing their morphology (arrowheads), stromal enrichment and disorganization of acinar tissue. (G) End-stage pancreatic adenocarcinoma with mixed acinar/ductal features showing an expansion of acinar tissue (black asterisk), its disorganization and duct hyperactivity (white asterisk). (H) Control exocrine pancreas Alcian Blue-negative samples showing organized acinar and duct structure. (I) End-stage mucinous Alcian Blue-positive pancreatic adenocarcinoma with goblet cells and small duct-like structures interspersed in a disorganized acinar tissue. Tumors shown in D, E, F and I were seen in 7 out of 15 analyzed samples between 3 and 5 mpf. Tumor shown in G was seen in 8 out of 15 analyzed samples between 3 and 5 mpf. (B′,D′,E′) More detailed magnifications of B (right), D and E. Arrowheads indicate acinar cells in B′, goblet cells in D′ and duct-like structures in E′. Age of analyzed samples is indicated. Scale bars: 50 μm.

Our second aim was the phenotypic characterization of collected fish: 120 *Tg(ptf1a:Gal4)/UAS:eGFP-KRAS^G12D^* injected individuals and 75 *Tg(ptf1a:eGFP)* controls. We focused our attention on fish survival and appearance of severe or lethal signs of disease. The observation time ranged from 1 to 100 weeks post fertilization (wpf). As reported in Fig. 1C, we found one peak of death at 1–2 wpf and two peaks of sickness at 4–6 wpf and 15–32 wpf for *Tg(ptf1a:Gal4)/UAS:eGFP-KRAS^G12D^*. Thus, about 92% of *Tg(ptf1a:Gal4)/UAS:eGFP-KRAS^G12D^* injected fish showed mortality and severe disease signs between 2 and 32 wpf, whereas the remaining 8% survived. *Tg(ptf1a:eGFP)* controls showed two peaks of mortality: the first at 1–2 wpf and the second at 72–100 wpf, showing a normal survival pattern. The phenotypic differences between *Tg(ptf1a:Gal4)/UAS:eGFP-KRAS^G12D^* injected and *Tg(ptf1a:eGFP)* were statistically significant according to uncoupled two-tailed Student’s *t*-tests performed on both groups. In order to understand the reason for three different peaks of lethality for *Tg(ptf1a:Gal4)/UAS:eGFP-KRAS^G12D^* injected fish, we made a more detailed observation. In particular, we observed that 60 out of 120 (50%) samples died after 2 wpf at larval stage; 35 out of 120 (30%) got sick at juvenile stage, showing strong motility disruption and swimming defects before dying; 15 out of 120 (12%) were collected at early adulthood, between 3 and 6 months post fertilization (mpf), as soon as they showed big protruding masses in the belly, later demonstrated to be pancreatic cancer (as shown in Fig. 2); and 10 out of 120 (8%), expressing *eGFP-KRAS^G12D^* only in cerebellum, reached adulthood without any alteration. Histological analysis of cerebellum from 35 out of 120 fish demonstrated that severe motility defects at 3–6 wpf, also related to a strong decrease in survival, were linked to a cerebellar dysplasia or to exacerbating medulloblastoma, resembling the pediatric form of human cerebellar cancer (shown in supplementary material Fig. S1).

### Histological features of pancreatic tumors

We followed pancreatic tissue growth by observing the progressive increase in eGFP fluorescence under control of *ptf1a* promoter elements. To characterize eGFP-positive pancreas extracted from *Tg(ptf1a:eGFP)* and *Tg(ptf1a:Gal4)/UAS:eGFP-KRAS^G12D^* injected samples, we performed histological analysis with hematoxylin-eosin (H&E) assay. As previously reported (Wan et al., 2006), we found that normal exocrine pancreas from all *Tg(ptf1a:eGFP)* fish is mainly characterized by arborized clusters of pancreatic acini surrounded by adipose tissue and flanked by bowel loops and liver (Fig. 2A). We collected 35 out of 120 *Tg(ptf1a:Gal4)/UAS:eGFP-KRAS^G12D^* injected samples between 2 and 5 wpf because of strong motility defects. All 35 fish expressed eGFP-KRAS^G12D^ in the cerebellum whereas only 16 out of 35 showed small focal eGFP-KRAS^G12D^-positive lesions in the exocrine pancreas, which was enlarged compared with normal tissue. We performed histological analysis on the 16 small abdominal eGFP-KRAS^G12D^-positive focal lesions to discriminate pancreatic lesions induced by KRAS^G12D^ during the first stages of carcinogenesis. Acinar hyperplasia, an early lesion observed during the development of pancreatic tumors showing an acinar phenotype, was seen in 6 out of 16 samples. Acinar hyperplasia is characterized by quite-organized, although abnormally abundant, acinar tissue that almost totally replaced the duct ephitelium (Fig. 2B left panel, 2C). The other ten animals also showed stromal enrichment progressively destroying the compact structure of exocrine tissue, becoming fibrous such as in acute pancreatitis (Fig. 2B right panel, 2B′). All 15 *Tg(ptf1a:Gal4)/UAS:eGFP-KRAS^G12D^* injected fish collected between 14 and 24 wpf showed widespread and protruding abdominal eGFP-KRAS^G12D^-positive masses in the gut region (supplementary material Fig. S2A,B). Histological analysis showed defined features of malignancy, such as the invasion of normal pancreas and surrounding organs. These tumors displayed a wide degree of heterogeneity with respect to histological patterns of differentiation, including pancreatic adenocarcinoma with acinar phenotype, pancreatic adenocarcinoma with mixed acinar and ductal features as already described (supplementary material Fig. S2A′,B′) (Park et al., 2008). In particular, we found that all 15 analyzed tumors showed a predominant acinar and mucinous phenotype and non-ductal differentiation. Out of 15 tumors, 7 displayed dramatic enrichment of stroma that infiltrated duct lumen, formation of strong reactive ductal-like structures and increased mucinous features, such as the presence of goblet cells similar to those observed in human and mouse pancreatic mucinous adenocarcinoma of acinar origin (Fig. 2D–F). These mucinous features were also demonstrated by staining pancreatic slices with Alcian Blue (Fig. 2H,I). The other eight samples revealed pancreatic adenocarcinoma of predominant acinar phenotype with mixed acinar/ductal features, mainly characterized by disorganized proliferation of cells with recognizable acinar morphology and an increasing number of ductal-like structures inside the exocrine pancreas (Fig. 2G).

To explore the reason for strong motility defects and decrease in survival of juveniles between 3 and 6 wpf, we performed H&E staining on 35 out of 120 fishes expressing eGFP-KRAS^G12D^ in cerebellum. As expected by eGFP-KRAS^G12D^ tissue-specific expression driven by *ptf1a*, we found the hallmark features of an undifferentiated medulloblastoma in both the external granular layer and ventricular zone in 5 out of 35 samples analyzed at 1 mpf (Aldinger and Elsen, 2008; Pascual et al., 2007). In contrast to control cerebellum of *Tg(ptf1a:eGFP)* fish (supplementary material Fig. S1A,B), the tumor appears as sheet-like areas of small, round, blue cells with scant cytoplasm and dense hyperchromatic nuclei (supplementary material Fig. S1C,D), resembling classic human cerebellar cancer (supplementary material Fig. S1E,F), which originates from granule cell progenitors located in the external granular layer (EGL) of the cerebellum (Marino, 2005). The other 30 collected samples, regularly expressing eGFP-KRAS^G12D^ in cerebellum, showed a normal histology with a few interspersed cells containing dense hyperchromatic nuclei, as seen in cerebellum dysplasia (data not shown).

### Activation of EMT, proliferation and apoptosis during pancreatic cancer progression

Cell hyperproliferation inside tumor lesions, low degree of apoptosis and changes in the tumor microenvironment (such as inflammation burst, neoangiogenesis, increase in cell stemness and EMT) are the main hallmarks of pancreatic cancer progression (Bardeesy and DePinho, 2002; Hanahan and Weinberg, 2011). In order to analyze some of these, we performed histology and immunohistochemistry (IHC) assays on 7 out of 15 collected pancreatic tumors.

**Fig. 3.**
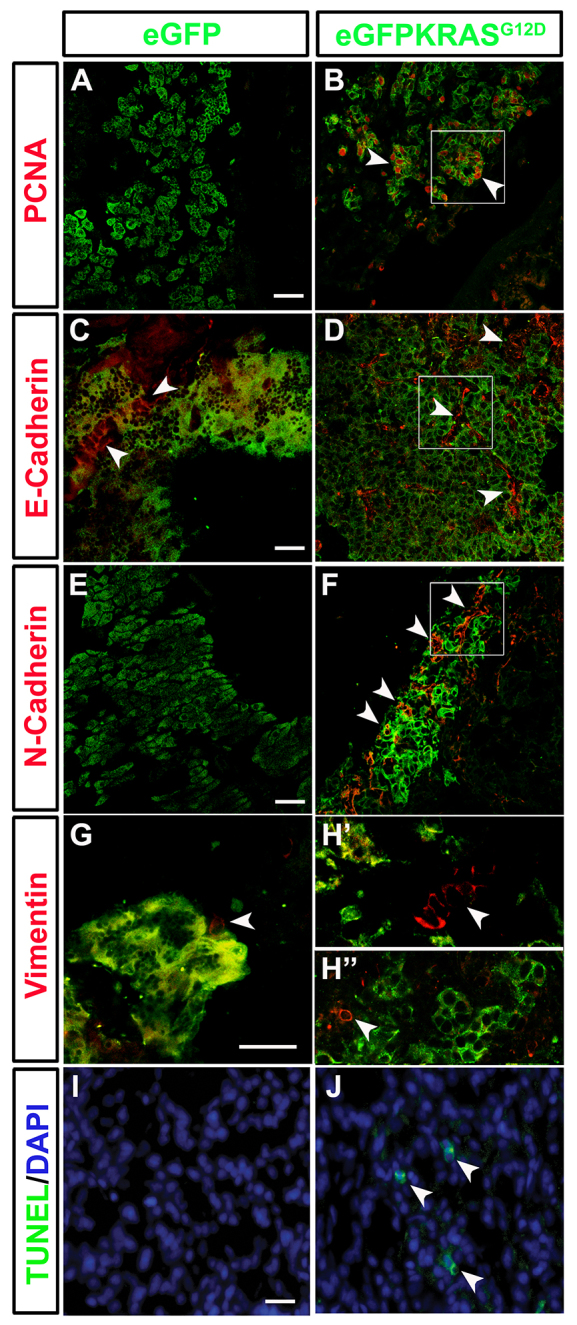
**Cell hyperproliferation, EMT and apoptosis induction during pancreatic cancer.** Markers of EMT (E-Cadherin, N-Cadherin and Vimentin), cell proliferation (PCNA) and apoptosis were assessed by immunohistochemistry on pancreatic tissue of both control *Tg(ptf1a:eGFP)* and tumor-prone *Tg(ptf1a:Gal4)/UAS:eGFP-KRAS^G12D^* lines. (A,B) The cell proliferation marker PCNA (white arrowheads) is increased in tumor-prone fish (B) compared with controls (A). Cropped image evidenced by white square is reported in supplementary material Fig. S3B′. (C,D) No differences in E-Cadherin (white arrowheads) expression was shown between pancreas of tumor-prone fish (D) compared with controls (C). Cropped image evidenced by white square is reported in supplementary material Fig. S3D′. (E,F) N-Cadherin (white arrowheads) is highly expressed in *Tg(ptf1a:Gal4)/UAS:eGFP-KRAS^G12D^* fish (F) whereas it is almost absent in controls (E). Cropped image evidenced by white square is reported in supplementary material Fig. S3F′. (G–H″) A slight increase in Vimentin expression (white arrowheads) was seen in both tumor mass and stroma of *Tg(ptf1a:Gal4)/UAS:eGFP-KRAS^G12D^* fish (H′,H″) whereas it resulted low expressed in controls (G). Apoptosis level was higher in tumor-prone fish (J) than in controls (I) as indicated by white arrowheads in J. Analyses were performed on 7 tumor and 7 control samples. All samples were analyzed at 3 mpf. Scale bars: 100 μm.

**Fig. 4.**
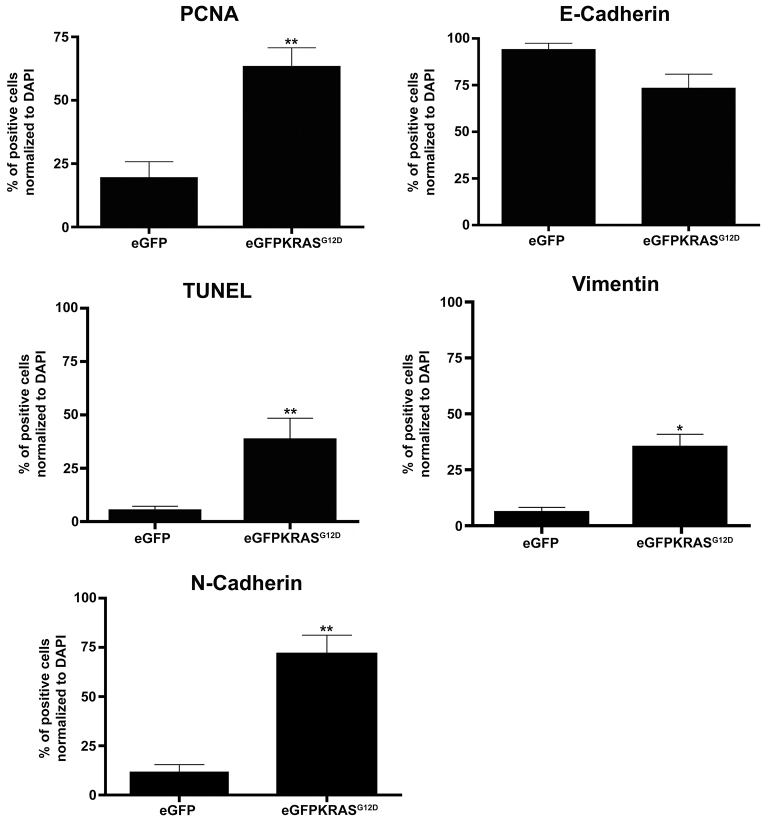
**Significant changes in levels of mesechymal, apoptosis and proliferation markers.** Quantification of IHC results by counting double-positive cells expressing eGFP-KRAS^G12D^ and PCNA, E-Cadherin, TUNEL, N-Cadherin or Vimentin in both *Tg(ptf1a:eGFP)* and *Tg(ptf1a:Gal4)/UAS:eGFP-KRAS^G12D^*. Results are reported as percentage of double eGFP-KRAS^G12D^/PCNA, eGFP-KRAS^G12D^/E-Cadherin, eGFP-KRAS^G12D^/TUNEL, eGFP-KRAS^G12D^/N-Cadherin and eGFP-KRAS^G12D^/Vimentin-positive cells normalized to DAPI counts. Results from 7 *Tg(ptf1a:Gal4)/UAS:eGFP-KRAS^G12D^* samples were all statistically significant, compared with 7 control *Tg(ptf1a:eGFP)* samples, except for E-Cadherin values. Error bars indicate s.e.m. for all analyzed samples; *P<0.05, **P<0.01 according to uncoupled Student’s *t*-test.

To understand the progressive enlargement of eGFP-KRAS^G12D^-positive masses, we assessed cell proliferation by using an antibody that specifically labels the PCNA (proliferating cell nuclear antigen) protein, a specific marker for cells in S phase of the cell cycle. Significantly higher expression levels of PCNA were seen in eGFP-KRAS^G12D^-positive masses, confirming a robust cell hyperproliferation during tumor development (Fig. 3A,B, Fig. 4; supplementary material Fig. S3B′).

In order to specifically label mesenchymal and epithelial cells, we analyzed the expression of E-Cadherin, N-Cadherin and Vimentin. The E-Cadherin to N-Cadherin switch is linked to the increase in mesenchymal features and is also involved in the mechanism of EMT (Nakajima et al., 2004; Rhim et al., 2012). It has been well described in pancreatic tumor progression and also during transdifferentiation processes such as the acinar to ductal metaplasia (Wu et al., 2012). E-Cadherin is a transmembrane protein involved in cell-cell adhesion. Whereas normally produced in acinar tissue of *Tg(ptf1a:eGFP)*, we observed a slight decrease in E-Cadherin expression in acinar tissue of *Tg(ptf1a:Gal4)/UAS:eGFP-KRAS^G12D^* injected fish (Fig. 3D). As shown in Fig. 4, reduction in the expression of E-Cadherin was not statistically significant in the pancreatic adenocarcinoma, possibly due to the normal levels of expression in the duct and endothelial cells, which hindered the differences between the exocrine pancreas and tumor mass (Fig. 3D; supplementary material Fig. S3D′).

N-Cadherin is a transmembrane protein mainly expressed during embryonic development and commonly found in cancer cells. It supports a mechanism of transendothelial migration leading to metastasis (Goonesinghe et al., 2012; Lirdprapamongkol et al., 2012; Scanlon et al., 2013). IHC analysis showed a statistically significant increase in N-Cadherin expression in eGFP-KRAS^G12D^-positive clusters, whereas its expression was totally absent in the pancreas of *Tg(ptf1a:eGFP)* controls (Fig. 3E,F, Fig. 4; supplementary material Fig. S3F′).

Analysis of mesenchymal markers was completed by observing the expression of Vimentin, the major cytoskeletal component of mesenchymal cells, previously used as the main sign of cells undergoing EMT (Savagner, 2010). We observed a Vimentin increase during pancreatic tumor progression in all 7 samples examined that were collected between 14 and 24 wpf (Fig. 3G,H′,H″). Not all Vimentin-positive cells are also eGFP-KRAS^G12D^-positive, indicating that the increase in mesenchymal cells is detected not only in tumor mass but also in the tumor stroma (Fig. 3H′). Thus, in agreement with previous data, we observed an increased expression of Vimentin in mesenchymal cells arising during cancer progression (Rhim et al., 2012; Satelli and Li, 2011). Except for the slight E-Cadherin decrease, all the results obtained comparing EMT and proliferation markers in eGFP-KRAS^G12D^-positive pancreatic tumors were statistically significant compared with control pancreas (Fig. 4).

Finally, to assess apoptosis we used the TUNEL (terminal deoxynucleotidyl transferase dUTP nick end labeling) assay. Compared with control *Tg(ptf1a:eGFP)*, we observed a significant increase in apoptosis in pancreatic adenocarcinoma of *Tg(ptf1a:Gal4;UAS:eGFP-KRAS^G12D^)* injected animals (Fig. 3I,J; Fig. 4).

### *In vivo* reporter analysis of the role of Shh, Notch and TGFβ in pancreatic tumors

Models of pancreatic adenocarcinoma have been useful in identifying genetic, molecular and biochemical processes regulating tumor progression (Salnikov et al., 2012). A wide variety of alterations in signaling pathways play pivotal roles in the pathogenesis of pancreatic tumors, affecting acinar or epithelial compartments together with the surrounding stromal microenvironment (McCleary-Wheeler et al., 2012). *KRAS* gene mutations are considered the main cause of pancreatic adenocarcinoma onset together with hyperactivation of the Shh signaling pathway (Jung et al., 2011; Park et al., 2008). Other molecular pathways involved during embryonic development, such as Notch and TGFβ, have been shown to be unbalanced in pancreatic adenocarcinoma but, for most, their specific role in tumor development remains to be investigated. We attempted to answer these questions by using specific transgenic lines *Tg(2xID1BRE:nlsmCherry)^ia17^; Tg(12xSBE:nlsmCherry)^ia15^; Tg(EPV.Tp1-Mmu.Hbb:nlsmCherry)^ia7^; Tg(12xGli-Hsv.Ul23:nlsmCherry)^ia10^*, reporting Bmp, TGFβ, Notch and Shh signaling pathways, respectively. All the lines were previously characterized for a correct reporter expression by using specific signaling inhibitors: LDN193189 for Bmp; SB431542 for TGFβ; DAPT for Notch; and cyclopamine for Shh (supplementary material Fig. S4). These lines were outcrossed with *Tg(ptf1a:Gal4)* and injected with *Tol2(UAS:eGFP-KRAS^G12D^)* as described in supplementary material Fig. S5. In order to identify which of these pathways are involved in pancreatic cancer onset, we performed confocal microscope analysis at 3, 5, 7, 30 and 60 days post fertilization (dpf), by using 10 eGFP-positive larvae from *Tg(ptf1a:eGFP)* and *Tg(ptf1a:Gal4)/UAS:eGFP-KRAS^G12D^* injected lines for each time point. Significance of data from each time point was confirmed by using the ANOVA test.

In agreement with previous evidence showing that deregulation of Wnt signaling occurs later in pancreatic tumor development and differentiation, linked to neo-vessels formation and tumor metastasis (Lowy et al., 2003; Morris et al., 2010), we did not find any Wnt/β-catenin reporter activity during pancreatic tumor onset (not shown).

**Fig. 5.**
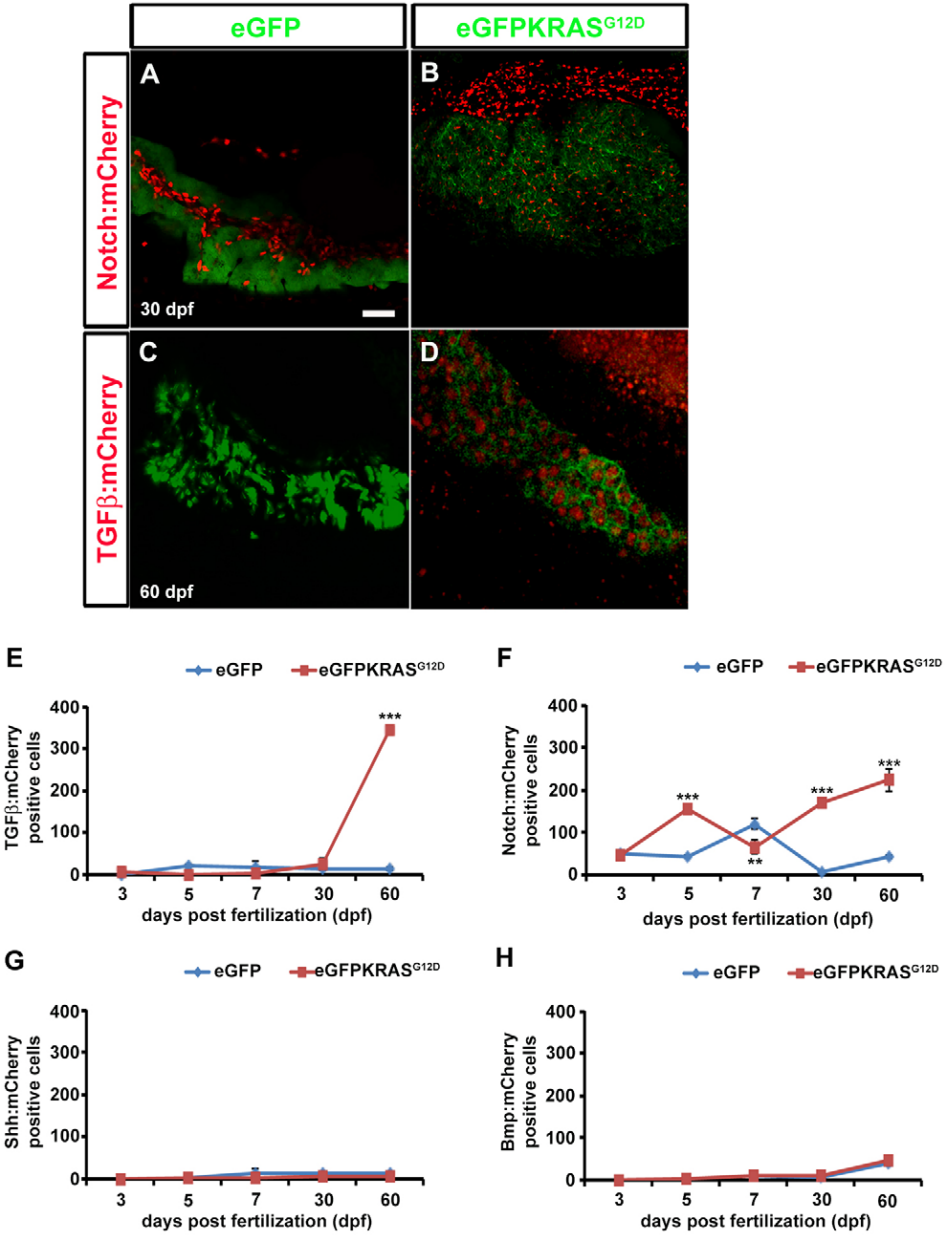
**TGFβ, Notch, Shh and Bmp signaling activities during pancreatic cancer onset.** TGFβ, Notch, Shh and Bmp signaling activities were evaluated by observing mCherry expression using confocal microscope analysis. (A–D) Pictures of full Z-stacks from 30 and 60 dpf samples. Controls obtained by outcrossing the *Tg(ptf1a:eGFP)* zebrafish line with TGFβ (A) and Notch (C) mCherry reporter lines. TGFβ (B) and Notch (D) signaling activity in tumor lines. (E–H) Quantitative analysis of eGFP-KRAS^G12D^-positive cells expressing TGFβ (E), Notch (F), Shh (G) and Bmp (H) mCherry reporters by VOLOCITY 6.0 software. For each time point, 10 eGFP-positive samples were analyzed. Results for TGFβ (E) and Notch (F) signaling were statistically significant. Error bars indicate s.e.m. for all analyzed samples; **P<0.01, ***P<0.005 according to ANOVA test.

We analyzed the Bmp signaling pathway, which is rarely involved in pancreatic tumor development, in an indirect manner, mediated by the PI3K/Akt pathway rather than Ras/MAPK (Chen et al., 2011; Handra-Luca et al., 2012; Virtanen et al., 2011). At 30 and 60 dpf, we did not observe any changes in Bmp signaling response in *eGFP-KRAS*^G12D^ fish compared with *Tg(ptf1a:eGFP)* controls (Fig. 5H). This result is in agreement with other studies, showing that Bmp is more needed in pancreatic development and differentiation during embryonic and larval stages than in tumor onset (Tiso et al., 2002; Xu et al., 2011).

We then verified whether Notch signaling, reported to be upregulated during both chronic inflammation and early stages of tumorigenesis, could play a main role (Su et al., 2006). We observed a strong increase in Notch reporter activity in acinar cells of the exocrine pancreas from *Tg(ptf1a:Gal4)/UAS:eGFP-KRAS^G12D^* injected animals (Fig. 5C,D) compared with *Tg(ptf1a:eGFP)* controls in which the reporter expression was totally ductal (Manfroid et al., 2012). By quantitative analysis, we documented a significant increase in Notch reporter activity at 5 dpf, which consistently remained upregulated at 60 dpf (Fig. 5F). Thus, our observation suggested an elevated Notch signaling activity during onset and progression of pancreatic cancer.

We next investigated the role of the Smad3/TGFβ signaling pathway by analyzing a specific mCherry reporter driven by Smad3 responsive elements; Smad3 is reported to be activated in pancreatic adenocarcinoma as a response to the antiproliferating function of TGFβ (Ungefroren et al., 2011). We observed a strong increase in TGFβ signaling responsive cells during pancreatic cancer onset between 30 and 60 dpf (Fig. 5A,B). In particular, *in vivo* analysis of the Smad3/TGFβ pathway in *Tg(12xSBE:nlsmCherry)^ia15^* fish at 2 mpf showed a strong increase in mCherry expression in three out of five fish. In these fish the reporter activity was clearly detectable in virtually all eGFP-KRAS^G12D^-positive cells, indicating a possible KRAS^G12D^-dependent activity for Smad2/3/4 (supplementary material Movie 1). Quantitative analysis of mCherry expression showed that the slight increase in TGFβ response seen in *Tg(12xSBE:nlsmCherry)^ia15^* fish was not significant at 30 dpf, whereas it started to be significant at 2 mpf (Fig. 5E). These data demonstrated that TGFβ is particularly involved during early stages of tumor development (Heldin and Moustakas, 2012; Truty and Urrutia, 2007). We also studied the expression of TGFβ at later stages of pancreatic tumor by performing the analyses on fish at 3–6 mpf. Our results confirmed the activation of the Smad3/TGFβ pathway, showing a strong increase in 12xSBE:mCherry expression rising at 16 wpf both in tumor mass (eGFP-KRAS^G12D^-positive cells) and stromal microenvironment (eGFP-KRAS^G12D^-negative cells) (supplementary material Fig. S6A,B; Movie 2).

Finally, during the early stages of carcinogenesis, the Shh reporter (i.e. the mCherry reporter linked to Gli1 responsive element) showed no Shh signaling until 30 dpf with a very slight increase at 2 mpf in tumor stroma (Fig. 5G).

**Fig. 6.**
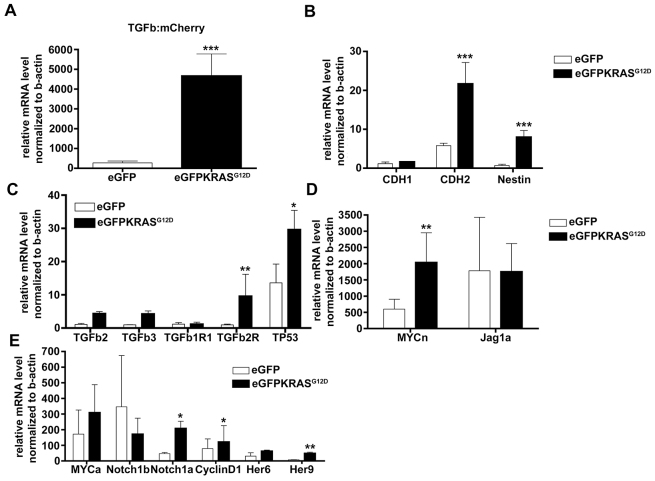
**Expression of genes and activation of pathways involved in pancreatic adenocarcinoma progression (real-time PCR data).** Real-time PCR assay was used to confirm IHC data. (A,B) Increasing mRNA levels of TGFβ:mCherry reporter (A), *cdh2* and *nestin* (B) were significant in *Tg(ptf1a:Gal4;UAS:eGFP-KRAS^G12D^)* compared with the control *Tg(ptf1a:eGFP*. (C–E) Additional real-time PCR experiments revealed significantly unbalanced expression of several genes including *tp53*, *TGFbR1b* (C), *notch1a*, *cyclinD1*, *her9* (D) and *MYCn* (E). Results are reported as mRNA level normalized to *ྞ-actin*. Error bars indicate s.e.m. for all analyzed data. Both Student’s *t*-tests and ANOVA tests were performed to statistically analyze data; *P<0.05, **P<0.01, ***P<0.005 comparing data between *Tg(ptf1a:Gal4;UAS:eGFP-KRAS^G12D^)* and *Tg(ptf1a:eGFP)*.

The involvement of the Smad3/TGFβ signaling pathway during pancreatic tumor progression was confirmed by real-time PCR, showing a significant increase (*P*<0.005) in *mCherry* mRNA levels in *Tg(ptf1a:Gal4)/UAS:eGFP-KRAS^G12D^* compared with the control (Fig. 6A). During later stages of pancreatic tumor, we also found a strong expression of specific genes related to stemness (e.g. *cdh2* (N-Cadherin) and *nestin*) (Fig. 6B), confirming the activation of EMT. Besides a significant increase in *p53* onco-suppressor and *TGFβR1* mRNA inside the eGFP-KRAS^G12D^-positive tumor mass (Fig. 6C), other pathways were directly or indirectly involved in pancreatic tumor progression: Notch and Wnt pathway activities increased at later stages of pancreatic tumor, as demonstrated by a significant increase in *notch1a*, *her9*, *myc* and *cyclinD1* mRNA levels (Fig. 6D,E).

In order to verify whether the unbalanced expression of Shh, Notch and TGFβ signaling pathways was similar in different tumors induced by *KRAS^G12D^*, we compared our reporter analyses on pancreatic adenocarcinoma with MDB data. In particular, to verify the reporter activities during MDB onset and progression, we analyzed eGFP-KRAS^G12D^ and mCherry expression by confocal microscopy at 3, 5, 7 and 30 dpf (supplementary material Fig. S7). We observed the inhibition of canonical Notch signaling and the increase in Smad3b-mediated TGFβ signaling at tumor onset (supplementary material Fig. S7). This is in agreement with results previously described in human and mouse models of MDB and mouse models of MDB (Aref et al., 2013; Guessous et al., 2008; Rodini et al., 2010). Smad3/TGFβ activity continued to be sustained till 30 dpf with a very slow decrease. Although we detected a general inhibition of canonical Notch signaling pathway during MDB development, basal Notch activity in KRAS^G12D^-positive cells was observed at 30 dpf (MDB later stages), suggesting that Notch might be involved in MDB progression (supplementary material Fig. S8). Sustained Smad3/TGFβ and Shh activities in KRAS^G12D^-positive cells were observed, underscoring the importance of these pathways in both onset and progression of MDB (supplementary material Fig. S6C,D; Movie 3). We also detected a rise in mesenchymal marker expression, as reported by significant increases in *nestin* and *notch1b* signaling together with a decrease in *gfap*, *olig4*, *olig2* and *ngn1*. Significant increase in *stat1b* and *TGFβ* signaling and a strong reduction in *p53* expression (supplementary material Fig. S9) were compatible with hyperproliferation of KRAS^G12D^-positive cells responsible for MDB progression (Rodini et al., 2010).

## DISCUSSION

In the first zebrafish model of pancreatic cancer, a mutant *KRAS* oncogene alone was able to induce pancreatic adenocarcinoma of predominantly acinar or mucinous phenotype (Park et al., 2008). To develop this cancer model, a transgenic BAC system expressing eGFP linked to oncogenic *KRAS^G12V^* in zebrafish pancreas under control of *ptf1a* promoter elements was generated. In this work, we were able to reproduce in zebrafish several KRAS^G12D^-dependent pancreatic cancers by using a conditional Gal4/UAS expression system. This strategy allowed us to follow pancreatic carcinogenesis both in space and time. Furthermore, we used the *KRAS^G12D^* rather than *KRAS^G12V^* mutated oncogene because of its major expression frequency in human cancers. *KRAS^G12D^* is also known to give rise to the most aggressive types of pancreatic adenocarcinomas and has a major role in cancer onset and maintenance (Rachagani et al., 2011). In agreement with the previously generated fish model, we reproduced pancreatic cancers displaying some similarities and some differences to both human and mouse models. Similarly to human and mouse, zebrafish pancreatic tumors showed a highly aggressive behavior and propensity for metastatic spread, as demonstrated by an increased expression of mesenchymal markers and infiltration of adjacent organs (Fig. 2 and Fig. 3), which ultimately led to the death of carrier animals. Unlike most human pancreatic cancers, however, we frequently observed features of non-ductal differentiation. In fact, both histological and immunohistochemical analyses confirmed a mixed acinar/ductal carcinoma of predominant acinar or mucinous phenotypes, becoming ductal at later stages of tumor development (Fig. 2) (Maitra and Leach, 2012; Park et al., 2008; Stanger et al., 2005). Moreover, Park et al. found that pancreatic progenitor cells expressing oncogenic *KRAS* under control of *ptf1a* promoter undergo normal specification and migration, but fail to differentiate (Park et al., 2008). This block in differentiation results in abnormal persistence of an undifferentiated progenitor pool. We confirmed this result by observing the upregulation of N-Cadherin and Vimentin mesenchymal markers (Figs 3, 4; supplementary material Fig. S3). Further, we found the upregulation of PCNA and p53 (Figs 3, 4, 6), demonstrating the cell hyperproliferation at the tumor site, a feature already reported in mouse and human tumors (Conradt et al., 2012; Ghosh and Leach, 2011; Gironella et al., 2007).

Moreover, we tried to unveil the most relevant molecular pathways involved in *KRAS*-mediated pancreatic tumor development by taking advantage of transgenic zebrafish reporter lines expressing responsive elements known to be pathway specific (supplementary material Fig. S4) (Moro et al., 2013). According to what has already been shown *ex vivo* on human pancreatic cancer (Bailey and Leach, 2012; De La O et al., 2008; Hu et al., 2012), we confirmed *in vivo* the upregulation of the Notch signaling pathway during tumor onset (Fig. 5), followed by the upregulation of the TGFβ/Smad3 pathway during tumor progression (Fig. 5; supplementary material Movies 1, 2). Nevertheless, the Notch pathway also remained active at later stages, possibly through the upregulation of Notch1a, as demonstrated by real-time PCR (Fig. 6) and confirming previous observations in human and mouse (Mysliwiec and Boucher, 2009; Wang et al., 2006b). Another evidence of TGFβ and Notch signaling upregulation at later stages of pancreatic adenocarcinoma was the concomitant upregulation of the gene encoding the TGFβ- and Notch-related target CyclinD1 (Fig. 6), involved both in neo-vessel formation and cancer metastases during the later stages of tumor development (Kornmann et al., 1999; Wang et al., 2006a; Wang et al., 2006b).

All described data demonstrate *in vivo* the leading role of the TGFβ pathway during mid and late stages of tumor progression. In particular, TGFβ started to increase at 1 mpf, reaching maximum expression at 2 mpf when mCherry reporter activity for TGFβ was detectable in most of the eGFP-KRAS^G12D^-positive cells (Fig. 5; supplementary material Movie 1). At later stages, as confirmed also by real-time PCR data (Fig. 6), levels of TGFβ signaling remained high but its upregulated activity was particularly observed in stromal cells surrounding the tumor (supplementary material Movie 2; Fig. S6). These two different spatiotemporal patterns of TGFβ expression confirm the idea that TGFβ has a double role: at early stages of carcinogenesis, the activity of TGFβ in most KRAS^G12D^-positive cells is likely to be post-mitotic, possibly acting as tumor suppressor; by contrast, at later stages, TGFβ expression in stromal cells surrounding the tumor could indicate a proinflammatory and oncogenic effect (McCleary-Wheeler et al., 2012). Furthermore, we observed high activity of the Notch pathway during pancreatic tumor onset, whereas Shh activity remained unaffected until 2 mpf when it started to be induced in a few eGFP-KRAS^G12D^-positive cells. These results demonstrate the progressive increase in TGFβ and Shh activities following KRAS^G12D^ constitutive activation during mid and advanced stages of pancreatic cancer progression, respectively.

Although there is little evidence demonstrating the involvement of mutated *KRAS* in MDB development (Gilbertson et al., 2006), we were able to reproduce a cancer model with features of pediatric MDB due to the specific expression of KRAS^G12D^ in *ptf1a*-expressing tissues (supplementary material Figs S1, S5–S9). Fish harboring medulloblastoma started to show strong motility disruption at 15 dpf and died at 45 dpf. To confirm the importance of TGFβ, Shh and Notch signaling pathways during carcinogenesis, we evaluated their activity during MDB onset and progression in parallel with pancreatic adenocarcinoma. As in pancreatic adenocarcinoma, we found a progressively increasing number of eGFP-KRAS^G12D^-positive cells activating TGFβ during MDB development. This result suggests that TGFβ is possibly regulated by the same mechanism, involving constitutive activation of *KRAS* oncogene during carcinogenesis of both pancreatic adenocarcinoma and MDB. Conversely, Notch and Shh signaling activities were observed to be different in the two cancers. In particular, Notch signaling was inhibited at early stages of MDB development, whereas in pancreatic adenocarcinoma, the co-activation of Notch and KRAS^G12D^ in acinar cells might be involved in acinar to ductal metaplasia during the early stages of carcinogenesis (De La O et al., 2008). We observed high Shh activity at MDB onset (supplementary material Movie 3; Fig. S6), confirming what is already known for human Shh-dependent MDBs (Bhatia et al., 2012). Notably, no Shh activity was seen during pancreatic adenocarcinoma onset. In other words, by comparing pancreatic adenocarcinoma with medulloblastoma, we were able to postulate that Smad3/TGFβ and Notch activities might be linked to the constitutive activation of KRAS^G12D^, the activity of Smad3/TGFβ and Notch reporters being present in KRAS^G12D^-expressing cells in both types of cancer. By contrast, Shh activity was upregulated in KRAS^G12D^-positive cerebellum but not in the pancreas where it was confined to the stroma of the pancreatic tumor at later stage of carcinogenesis, in agreement with previous observations (Park et al., 2008).

These results suggest that zebrafish cancer models, coupled with transgenic zebrafish reporter lines, are powerful tools for the analysis *in vivo* of the initiating events of pancreatic tumorigenesis and the sequences of hallmark progression during cancer development. Moreover, Notch, TGFβ and Shh ligands and their effectors could be interesting targets for high-throughput screening of drugs to be used in efficacious combined or sequential therapy of several malignant diseases such as MDB and pancreatic adenocarcinoma.

## MATERIALS AND METHODS

### Generation of transgenic reporter zebrafish lines for Shh, Bmp, TGFβ and Notch pathways

The generation of all reporter vectors was performed by using the Multisite Gateway-based construction kit (Tol2 kit), provided by Chi-Bin Chien of the University of Utah (USA) (Kawakami et al., 2004; Kwan et al., 2007). All sequences for the responsive elements were PCR amplified using oligos carrying the *Hin*dIII and *Bam*HI restriction sites and cloned into the p5EMCS vector. For generation of a transgene containing a TGFβ-responsive element, a (CAGA)_12_ box (also called 12xSBE for Smad3 binding element) was selectively amplified with adaptor oligos (Dennler et al., 1998). To generate a Notch reporter transgenic line, a Notch-responsive box (donated by Nathan Lawson, University of Massachusetts Medical School, Worcester, MA) containing 12× multimerized oligonucleotide sequences, corresponding to 12 copies of the Epstein Barr Virus terminal protein 1 (*TP1*) promoter (12 Rbp-Jκ binding sites), has been integrated into the zebrafish germline (Parsons et al., 2009). To obtain the vector carrying Bmp-responsive elements, a transcription cassette (−1046;−863) with the minimal promoter region and the TATA box of the human *ID1* gene was isolated from the plasmid pID183 (López-Rovira et al., 2002). The Shh-responsive construct is described elsewhere (Corallo et al., 2013). Each p5EMCS containing the responsive elements was recombined with middle pME-MCS vectors carrying the fluorescent protein coding sequences (GFP/mCherry). The final vectors containing the reporter were sequenced on both strands to verify the correct orientation of the responsive elements, minimal promoter, fluorescent protein coding sequence and SV40 poly-A. A pCS2FA-transposase construct necessary to generate transposase mRNA was included in the Tol2 Kit. To finally obtain the transgenic reporters lines, eggs derived from wild-type zebrafish were co-injected with transposase mRNA and reporter plasmids. Larvae were analyzed with fluorescence microscopy at different developmental stages. Stable lines were isolated from F1 carriers.

### Generation of pancreatic adenocarcinoma zebrafish model and analysis of molecular pathways involved in tumor onset

We injected *Tol2(UAS:eGFP-KRAS^G12D^)* plasmid into one-cell stage embryos derived from incrosses or outcrosses of *Tg(ptf1a:Gal4)^jh16^* zebrafish line with signaling mCherry reporter lines. Zebrafish reporter lines used in this work were *Tg(2xID1BRE:nlsmCherry)^ia17^*, *Tg(12xSBE:nlsmCherry)^ia15^*, *Tg(EPV.Tp1-Mmu.Hbb:nlsmCherry)^ia7^* and *Tg(12xGli-HSV.Ul23:nlsmCherry)^ia10^* (supplementary material Fig. S5). Approximately 150 embryos were raised, all expressing eGFP according to the expected *ptf1a* pattern. Transcutaneous eGFP expression was evaluated at 1-week intervals until development of a tumor mass, when fishes were euthanized for further histological and histochemical evaluation. Larvae at 3, 5, 7 dpf and 1 mpf were photographed live using a NIKON C2 H600L confocal microscope with 20× and 40× water dipping objectives. Lasers used to excite fluorophores were 488 nm for eGFP and 561 nm for mCherry. The number of single eGFP-KRAS^G12D^-positive cells also expressing the mCherry reporter was calculated using VOLOCITY 6.0 software (PerkinElmer, Waltham, MA) on eGFP-positive confocal acquired images. Embryos and larvae were anesthetized using Tricaine and mounted in 0.8% low melting agarose on a glass lid before photographing. The project, with protocol number 18746, was examined and approved by the Ethical Committee of the University of Padua.

### Histology and immunohistochemistry of tumors

Pancreas and cerebellum of eGFP-KRAS^G12D^-positive fish and *Tg(ptf1a:eGFP)* controls were dissected and fixed in PBS containing 4% paraformaldehyde overnight at +4°C. Collected tissues were paraffin-embedded, cut as 1 μm slices and examined histologically using the standard H&E and Alcian Blue methods to analyze cell morphology and tissue structure. Tissue slices were immunostained with DAPI, to label cell nuclei, and antibodies anti-E-Cadherin (ab53033, Abcam, Cambridge, UK), anti-N-Cadherin (ab12221, Abcam, Cambridge, UK), anti-Vimentin (M7020, Dako, Glostrup, Denmark), and anti-PCNA (M0879, Dako, Glostrup, Denmark), according to standard procedures. A TUNEL assay protocol (Invitrogen, Carlsbad, CA) was used to detect apoptosis.

### Quantitative RT-PCR and statistical analysis

Total mRNA was isolated from the pancreas and cerebellum of zebrafish embryos using Trizol (Invitrogen, Carlsbad, CA) and 0.5 μg of total RNA reverse-transcribed using SuperScript RNaseH-Reverse Transcriptase (Invitrogen, Carlsbad, CA). Quantitative RT-PCR reactions were run in triplicate using Brilliant^®^ SYBR^®^ Green QPCR Core Reagent Kit (Stratagene, La Jolla, CA). Fluorescent emission was recorded in real time (Sequence Detection System 7900HT, Applied Biosystems, Carlsbad, CA). Gene expression analysis was completed using the comparative Ct method of relative quantification (Spatuzza et al., 2008). PCR amplification conditions consisted of 40 cycles with primer annealing at 60°C. Sequences of specific primers used in this work are listed in supplementary material Table S1. Primers were designed using the software Primer 3 (http://bioinfo.ut.ee/primer3-0.4.0/input.htm). PCR amplicons were previously evaluated on agarose gel and, during SYBR green analyses, primer dissociation curves were checked in each run to ensure primer specificity on human and zebrafish mRNA. Relative RNA quantities were normalized to β-actin.

Statistical analyses to compare results for *Tg(ptf1a:Gal4)/UAS:eGFP-KRAS^G12D^* injected samples and *Tg(ptf1a:eGFP)* controls were performed using uncoupled Student’s two-tailed *t*-test and Microsoft Excel 2011 or Prism GraphPad software package. The ANOVA test was performed to analyze statistical differences in pathway expression at different time points of cancer development.

## Supplementary Material

Supplementary Material
