# Supplementary material for: Zebrafish reporter lines reveal in vivo signaling pathway activities involved in pancreatic cancer
Source: Dis Model Mech. 2014 May 30;7(7):883–94. doi: 10.1242/dmm.014969 (PMC4073277; doi:10.1242/dmm.014969)
Supplement: Supplementary Material [file supp_7_7_883__index.html]

Zebrafish reporter lines reveal in vivo signaling pathway activities involved in pancreatic cancer — Supplementary Material 

# Zebrafish reporter lines reveal *in vivo* signaling pathway activities involved in pancreatic cancer

## DMM014969 Supplementary Material

**Files in this Data Supplement:**

- **Supplementary Material**
