# Supplementary material for: Zebrafish reporter lines reveal in vivo signaling pathway activities involved in pancreatic cancer
Source: Dis Model Mech. 2014 May 30;7(7):883–94. doi: 10.1242/dmm.014969 (PMC4073277; doi:10.1242/dmm.014969)
Supplement: Supplementary Material [file supp_7.7.883_DMM014969.pdf]

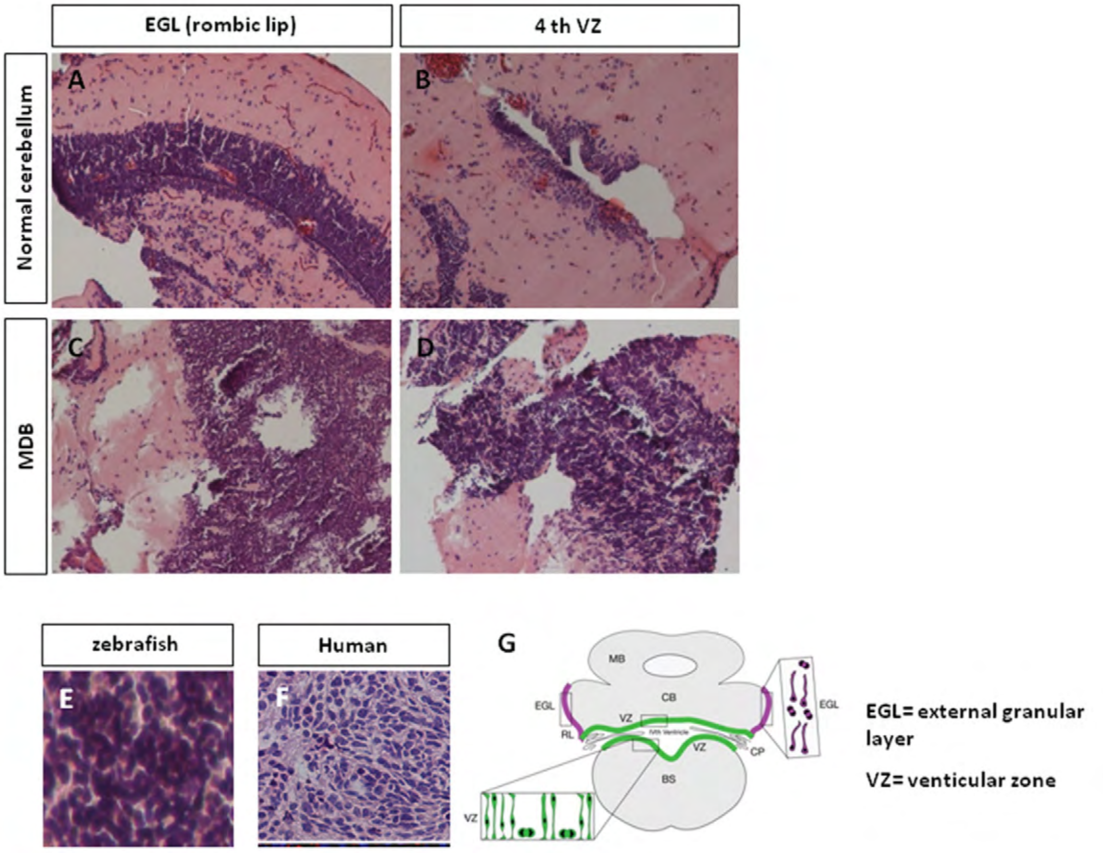

Fig.S1. ***KRAS**^{G12D}* **overexpression in zebrafish cerebellum induces medulloblastoma (MDB). A-D**: Appearance of EGL (**A**) and 4th VZ (**B**) in normal cerebellum; zebrafish injected with *KRAS^{G12D}* develop early cerebellar lesions (**C,D**), which resemble medulloblastoma, starting from 15 dpf. **E, F**: Analysis of the histological features in 1 mpf zebrafish cerebellum reveals tumour masses (**E**) that resemble the classic type of human medulloblastoma (**F**) with sheets of small round blue cells with scant cytoplasm. (**G**) Schematic representation of a normal cerebellum, with EGL and VZ locations.

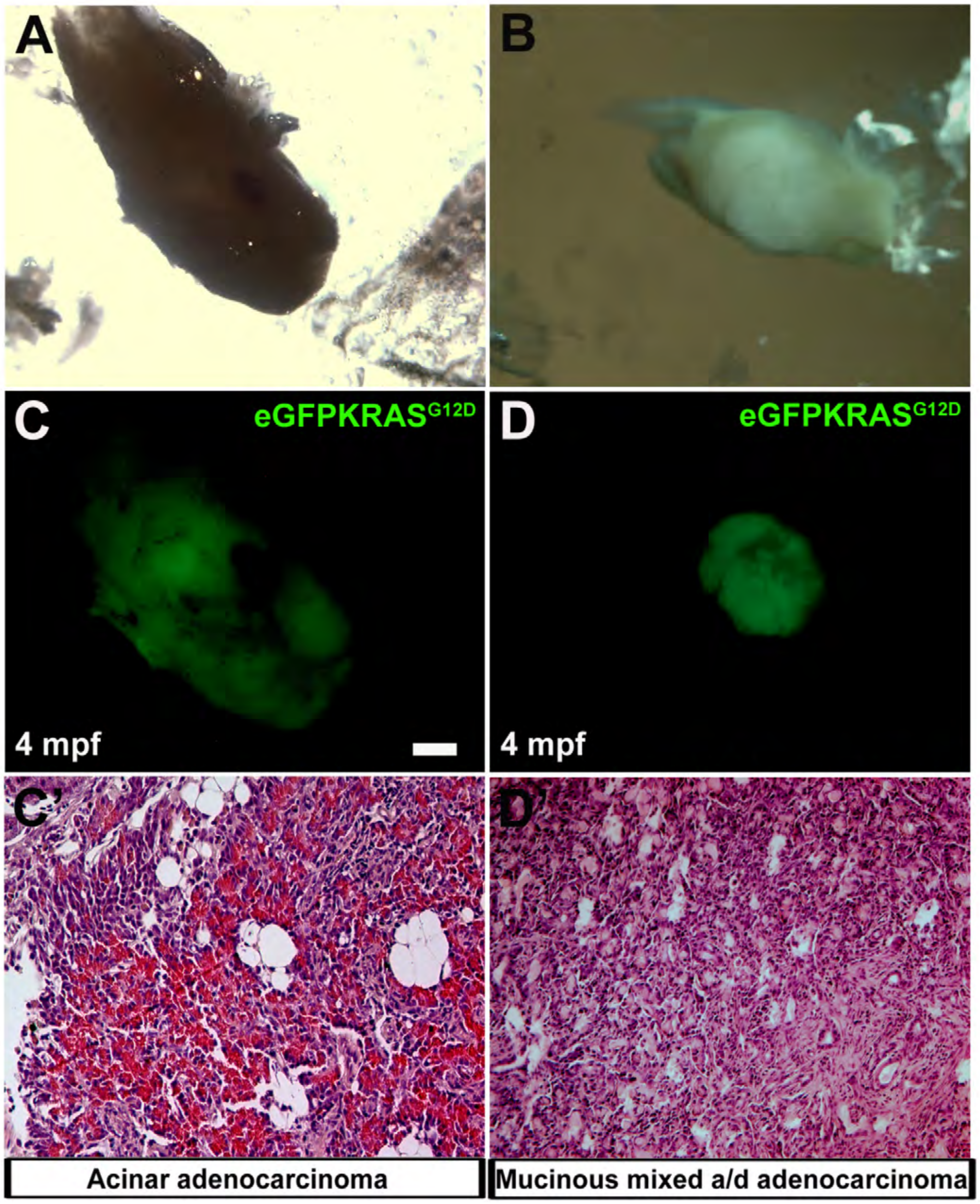

Fig.S2. Different types of pancreatic cancers. In the panels **A** and **B** are reported brightfield images of two different excised pancreatic tumors at 4 months post fertilization. In the panels **C** and **D** are reported the relative eGFPKRAS^{G12D} positive tumor masses. Two masses corresponded to different types of pancreatic adenocarcinomas: acinar adenocarcinoma with altered structure of exocrine tissue (**C'**) and mucinous mixed acinar/ductal (a/d) adenocarcinoma (**D'**). Scale bar is 50 **µm**.

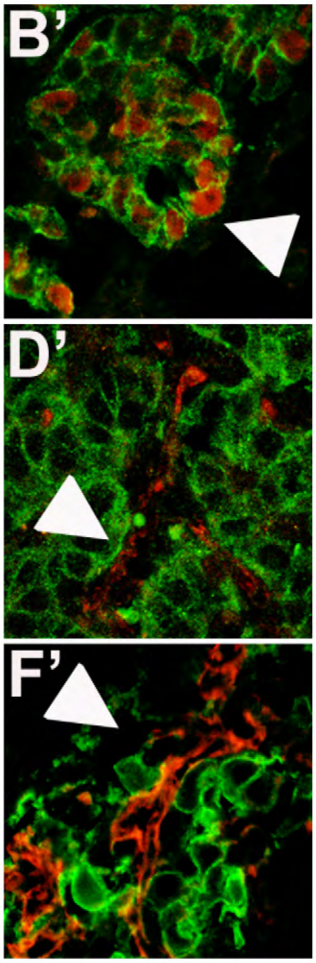

Fig.S3. Cropped images from Fig.3: Cropped images evidenced in white squares of **Fig.3** for PCNA (**B'**), E-Cadherin (**D'**), N-Cadherin (**F'**) expression in eGFP positive tumor masses.

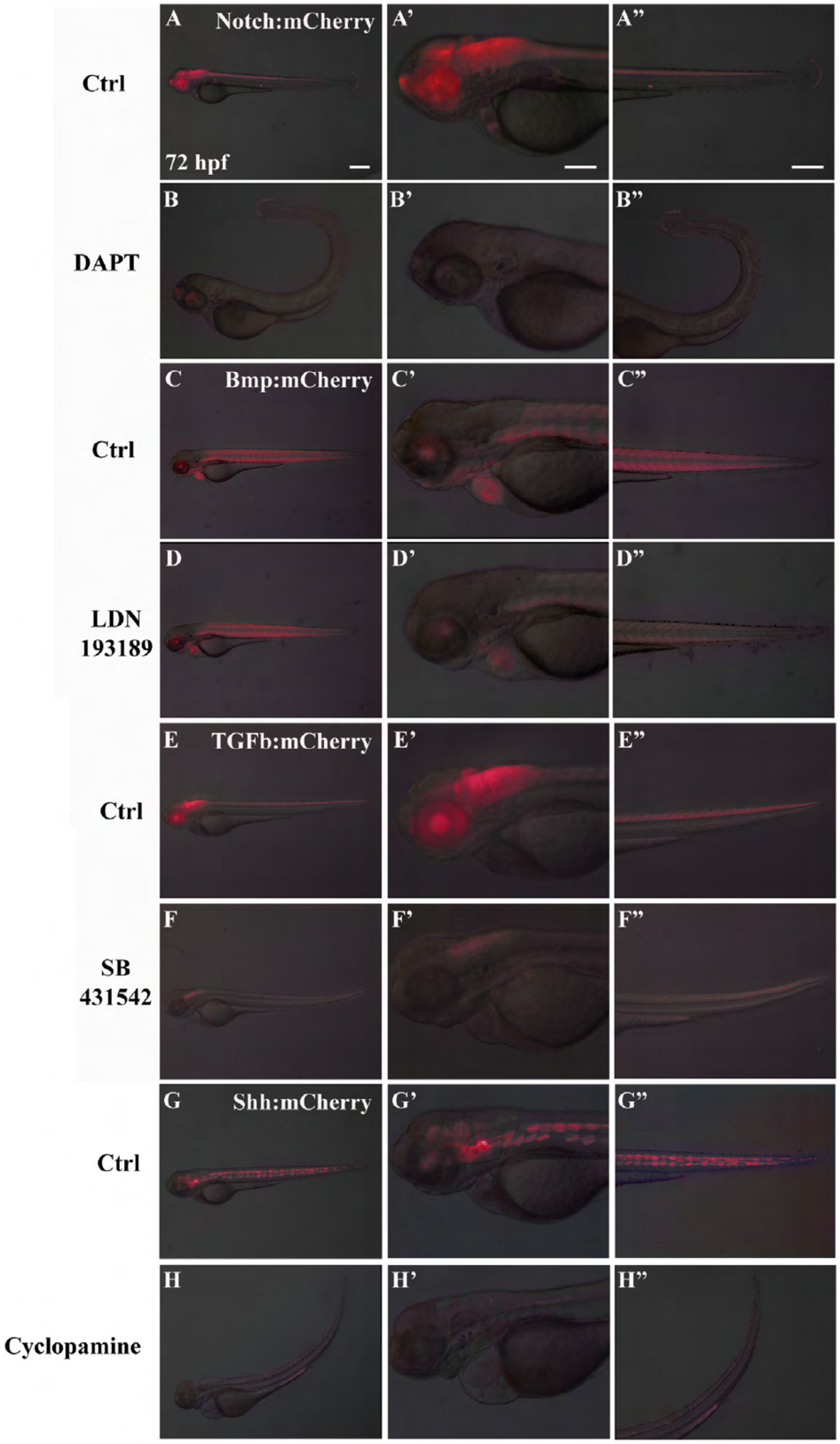

Fig.S4. Validation of mCherry reporter expression. mCherry was expressed under control of ID1 (Bmp signaling); Smad3 (TGFβ signaling); Gli1 (Shh signaling) and Rbpj (Notch signaling). Panels report embryo analysis at 72 hpf for both treated and untreated embryos. Notch signaling is expressed in entire neural tube, pineal gland, eyes, cardiac valves and tail (**A-A"**); Bmp signaling is expressed in muscle fibers, eyes, gill arches, heart (**C-C"**); TGFβ signaling is expressed in neural tube except midbrain and forebrain and eyes (**E-E"**); Shh signaling is expressed in nothocord, midbrain, hindbrain and eyes (**G-G"**). Embryos were treated with specific pathways inhibitors: DAPT for Notch signaling (panels **B-B"**), LDN193189 for Bmp signaling (panels **D-D"**), SB431542 for TGFβ signaling (panels **F-F"**), Cyclopamine for Shh signaling (panels **H-H"**). **A'–H'** and **A"–H"** are magnifications of **A-H.** Scale bar is 50 μm for all pictures. Images are representative. Even considering the initial variability of reporter expression, we could observe significant reduction (more than 75%) of the fluorescence in all embryos analyzed for each reporter line treated with specific inhibitors (n>100/line).

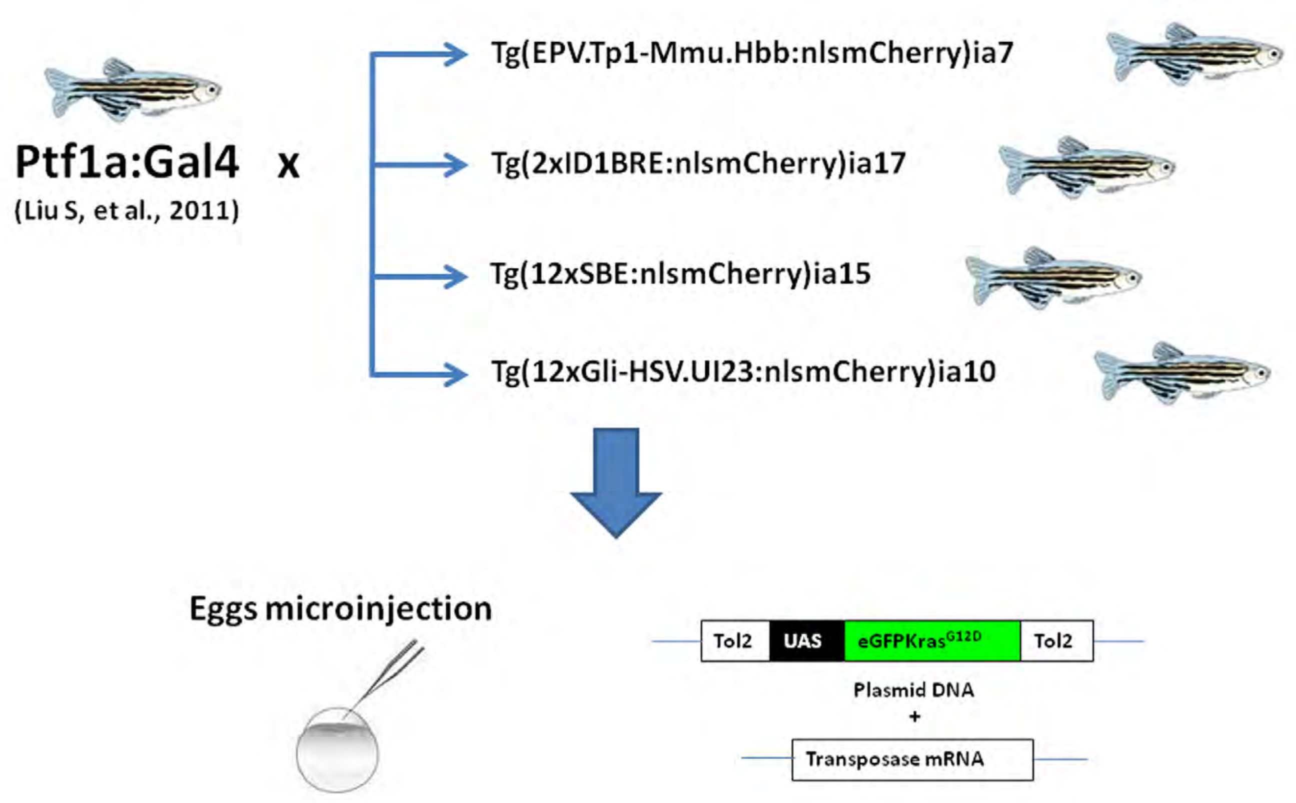

Fig.S5. Generation of pancreatic adenocarcinoma zebrafish model. To generate our model of pancreatic adenocarcinoma, we used the conditional Gal4/UAS expression system. The *Tg(Ptf1a:Gal4)* driver line, expressing Gal4 in pancreas and cerebellum under the control of tissue-specific *ptf1a* promoter, was outcrossed with *Tg(EPV.Tp1-Mmu.Hbb:nlsmCherry)*^{ia7} for Notch signaling; *Tg(2xID1BRE:nlsmCherry)*^{ia17} for BMP signaling; *Tg(12xSBE:nlsmCherry)*^{ia15} for TGFβ signaling; *Tg(12xGli-HSV.UI23:nlsmCherry)*^{ia10} for Shh signaling. For Tol2-mediated transgenesis, the fertilized eggs, derived from these outcrosses, were co-injected with Tol2 transposase mRNA and a Tol2 plasmid, harboring the *eGFPKRAS*^{G12D} transgene under control of Gal4 responsive elements (UAS).

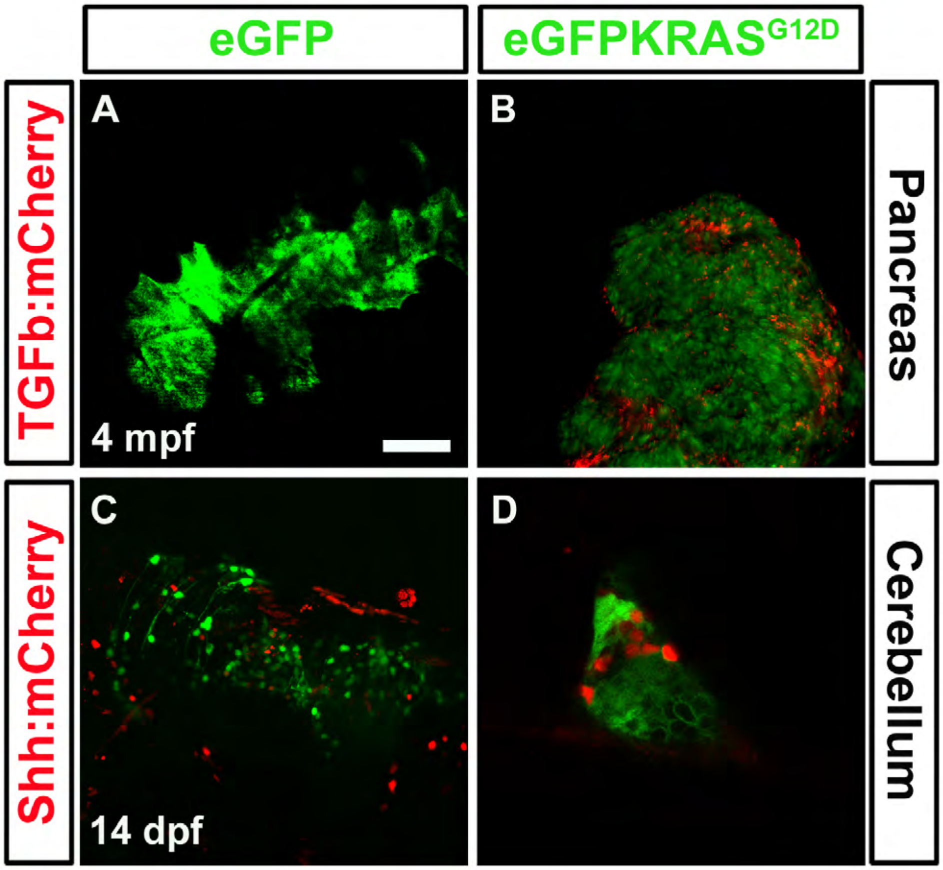

Fig.S6. Role of TGFβ and Shh pathways during cancer progression. Panels **A** and **C** represent the *Tg(ptf1a;eGFP)* controls while panels **B** and **D** represent eGFPKRAS^{G12D} positive tumor masses. High levels of TGFβ:mCherry expression in the stroma around pancreatic tumor mass at 4 mpf are depicted in panel (**B**). Shh:mCherry is highly expressed in cerebellum at 14 dpf (**D**). All figures are single confocal Z-stack planes. Normal tissues and tumor masses are in **green**, while activity of Shh and TGFβ are in **red**. Scale bar is 50 μm.

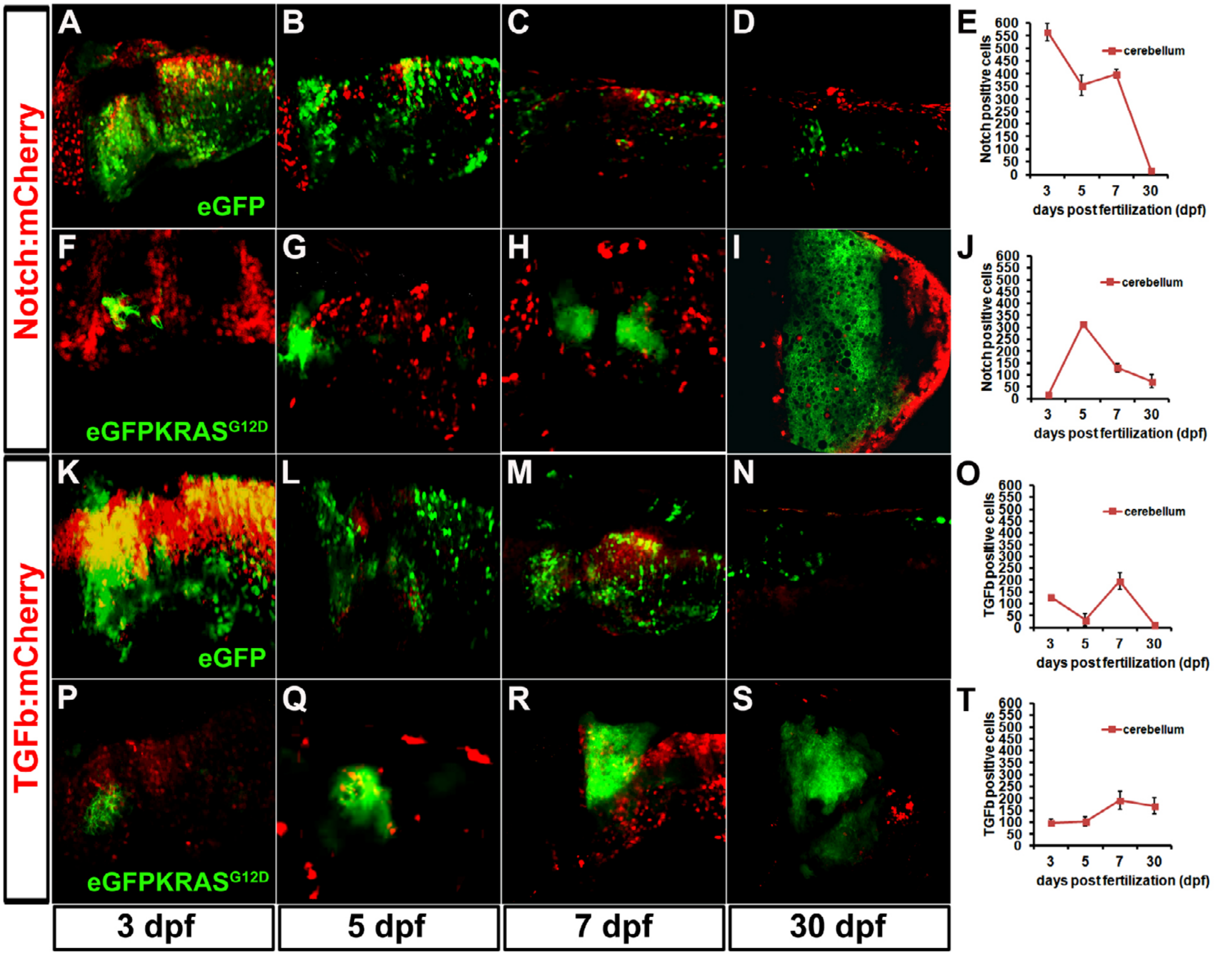

Fig.S7. Role of Notch and TGFβ pathways during MDB onset and progression. Confocal analysis was performed to dissect the role of Notch and TGFβ pathways during MDB development. **A-D:** activity of Notch signaling pathway (**red signals**) in normal cerebellum (**in green**). **E**: Graph displaying the strong decrease of Notch:mCherry signal during development. **F-J:** inhibition of canonical Notch pathway (**red signals**) during MDB onset (3 - 7 dpf; **F-H**) and very low activity during MDB progression (30 dpf; **I**), as depicted also in graph (**J**)**,** in parallel with the strong enlargement of eGFPKRAS^{G12D} tumor masses (**green signals**). Panels **K-N** display the activity of TGFβ signaling pathway (**red signals**) in MDB-free conditions. The alternating behavior of TGFβ:mCherry reporter line is depicted in graph (**O**). **P-T**: Increase of TGFβ signaling pathway (**red signals**) in MDB onset (3–7 dpf; **P-R**) and its involvement during MDB progression (**S**), as also depicted in graph **T**. Scale bar in all images is 50 **µm**, as reported in panel **A.** T student analysis showed P < 0.005, indicating that differences between MDB and control samples are statistically significant. Error bars are reported in each graph. Results in **E, J, O, T** are reported as eGFP/mCherry positive cell number.

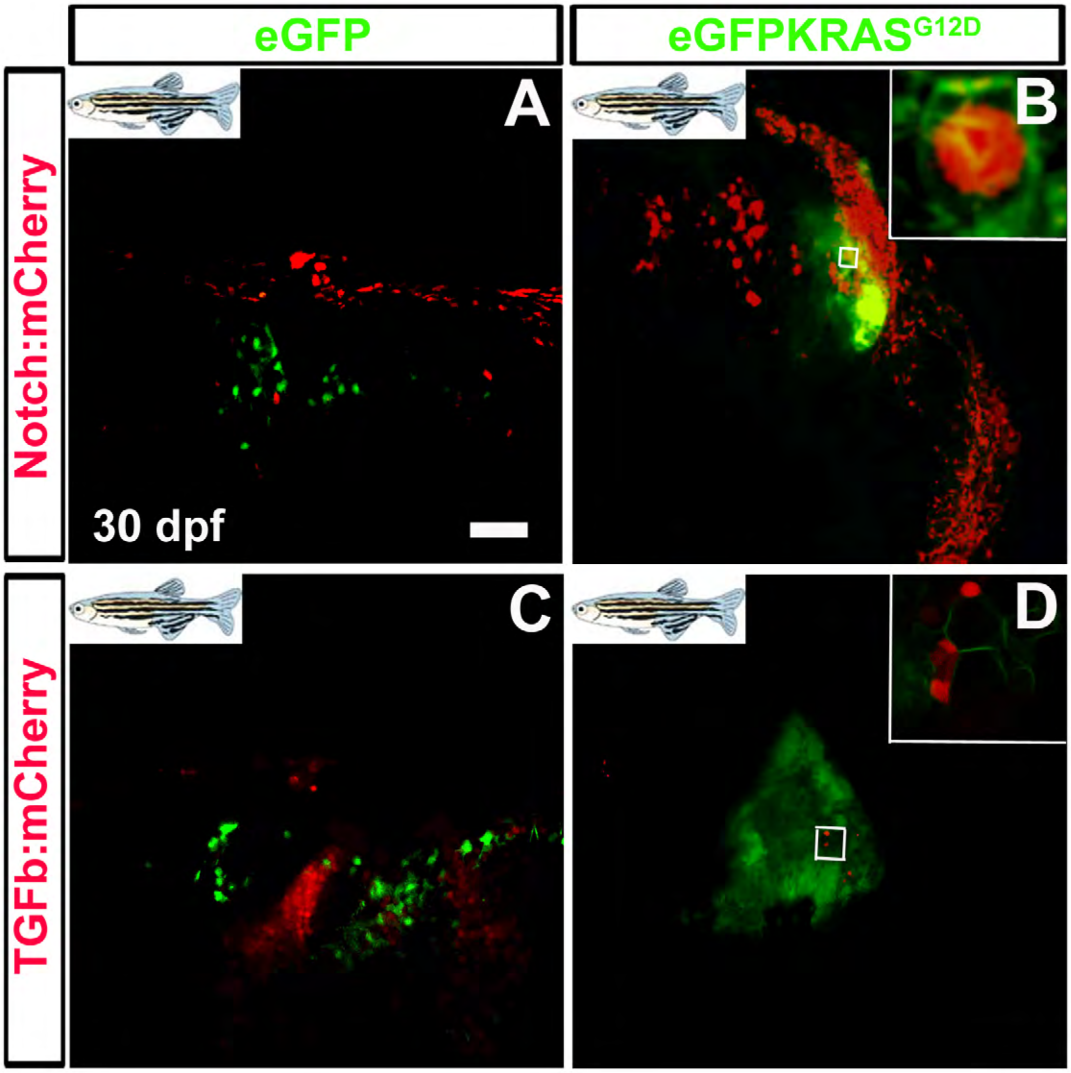

Fig.S8. *KRAS* positive cells expressing Notch and TGFβ signaling reporters during MDB progression. Confocal zoom was performed on cerebellum tissue to observe the activity of TGFβ and Notch pathways at single-cell level. Normal (**A** and **C**) and Hyperplastic cerebella (**B** and **D**) are depicted, with scale bars for original (50 μm; **A, B, C, D**). The evidenced enlargement in panel **B** shows *KRAS* dependent activity of TGFβ pathway in MDB (tumor-prone green cells are also red reporter-positive). The enlargement in panel **D** shows *KRAS* dependent activity of Notch pathway in MDB (tumor-prone green cells are also red reporter-positive). Both pathways appear simultaneously involved in different ways during MDB onset and progression.

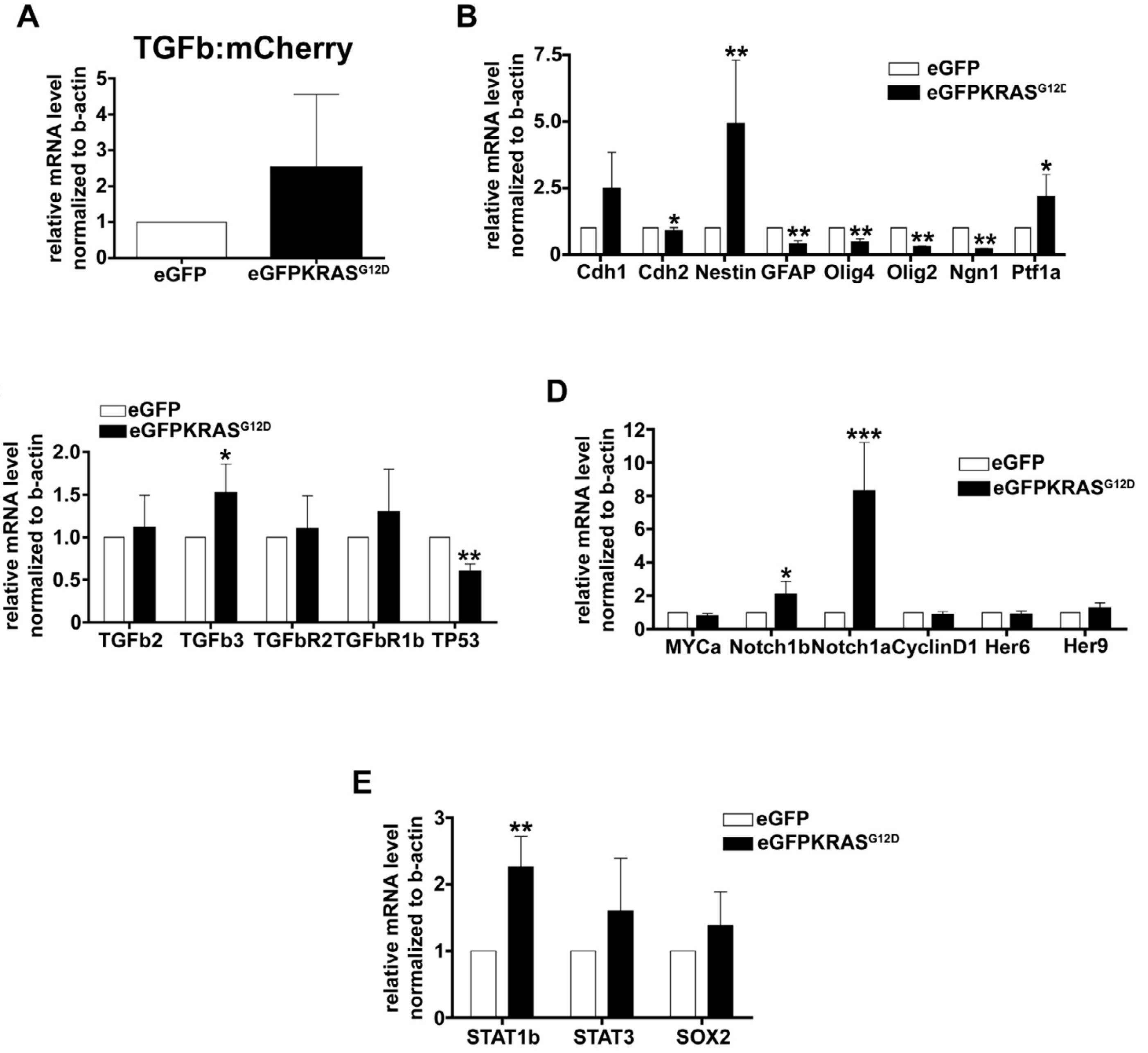

Fig.S9. Expression of genes and activation of pathways involved in medulloblastoma (Real-Time PCR data). Real Time PCR assay was used to confirm IHC data. **A:** mRNA level of TGFb:mCherry; **B**-**D:** mRNA levels of *nestin*, *gfap*, *olig4*, *olig2*, *ngn1*, *ptf1a*, *notch1b*, *notch1a*, *stat1b*, *TGFβ3*, *tp53* were significant in *Tg(ptf1a:Gal4)/UAS:eGFP-KRAS*^{G12D} compared to the control *Tg(ptf1a:eGFP)*. Significant (* for p<0.05; ** for p<0.01) and non significant results are obtained comparing data between *Tg(Ptf1a:Gal4)/UAS:eGFP-KRAS*^{G12D} and *Tg(Ptf1a:eGFP).* Results are reported as mRNA level normalized to *b-actin*. Error Bars are reported for all analyzed data.

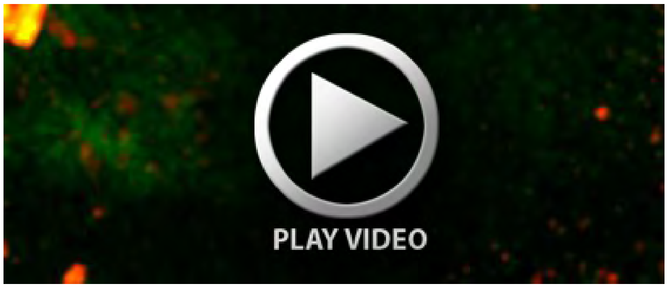

Supplemental Movie 1. Sustained cell-autonomous TGFβ signaling activity of $KRAS^{G12D}$ cells during first stages of pancreatic adenocarcinoma progression at 2 mpf. Z-stack confocal imaging of a 2 months old *Tg(Ptf1a:Gal4)/UAS:eG-FP-KRAS^{G12D};TGFβ:mCherry* fish. Lateral view of entire exocrine pancreas. All Z-stacks were collected together. Almost all eGFP-$KRAS^{G12D}$ cells (**green**) express the TGFβ:mCherry reporter (**red**).

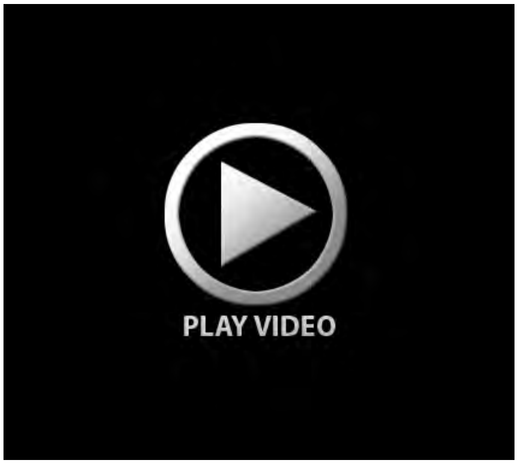

Supplemental Movie 2. Detection of stromal non cell-autonomous TGFβ signaling activity during pancreatic adenocarcinoma progression at 4 mpf. Z-stack confocal imaging of a pancreas dissected from a 4-month old *Tg(Ptf1a:Gal4)/UAS:eG-FP-KRAS^{G12D};12xSBE:mCherry* fish. The movie is composed of 1-75 Z-stacks collected together. The Smad3:mCherry reporter (**red)** appeared expressed all around the eGFP-$Kras^{G12D}$ tumor mass (**green**).

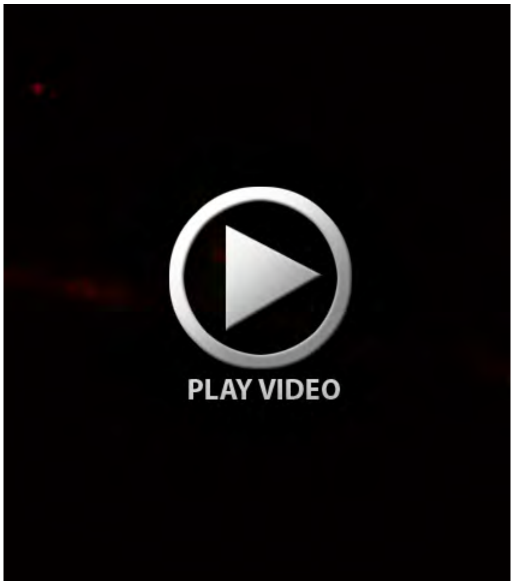

Supplemental Movie 3. Strong cell-autonomous Shh signaling activity during MDB onset at 14 dpf. Z-stack confocal imaging of a 2-weeks old *Tg(Ptf1a:Gal4)/UAS:eGFP-KRAS^{G12D};Shh:mCherry* tumor-prone fish larva. Lateral view of an entire cerebellum. The movie is composed of 1-55 Z-stacks collected together. The Shh:mCherry reporter (**red**) appeared to be expressed only in eGFP-$KRAS^{G12D}$ positive cells (**green**).

Table S1: List of primers used in Real Time (RT) PCR experiments.

**Table S1**	
**Sequence of primers used in RT-PCR**	
Gene	***Sequence (5'-3')***
Tgfb2 forward	GCAGCCCTTTTACCCTTCAG
Tgfb2 reverse	CGCCTTCACCAAGTTTGAGG
Tgfb3 forward	AGGGACGGATGAGTGGGTTT
Tgfb3 reverse	CTCCGTTGGGTCTGAAGGTG
Stat1b forward	ACGATGAGGCTTTCCCAATGG
Stat1b reverse	GCTCATGGAAGCGAACGATGG
Stat3 forward	GTTGGAGACGCGGTATCTGG
Stat3 reverse	CCCAGCAGGTTGTGGAAGAC
Pax6 forward	GGGCGCAGATGGCATGTATG
Pax6 reverse	CGCCTCCGTCTGACTGTTG
Nestin forward	AGAGCTGGAGAGCAGAGGA
Nestin reverse	TCCAGGGTGTTTACTTGGGC
Sox2 forward	CACCAACTCCTCGGGAAACA
Sox2 reverse	GTGCATTTTGGGGTTCTCCTG
Tgfbr2 forward	GCAACGACATGCTGCTGTTC
Tgfbr2 reverse	GAGTTTTCTGCGGCGGTACA
Tgfbr1b forward	CACAGAGTCGGCACCAAACG
Tgfbr1b reverse	GCGATCTCCCAGAACACCAG
Cdh1 forward	GAAGCACACGGCATCTGTCA
Cdh1 reverse	GGACCAGCCTCATTGGGAAT
Cdh2 forward	GCGGAGAGGAAGACCAGGA
Cdh2 reverse	TAGTTGGGCTCCGAGTGCAT
Myca forward	GTGGCAGCGATTCAGAAGATG
Myca reverse	CTCGTGCCTTTTCTGTCGCT
Mycn forward	CGTGTTTCTACCCGGACGAG
Mycn reverse	TCGGGGACAGAGGAGGAGT
mCherry forward	CCCTCAGTTCATGTACGGC
mCherry reverse	GGGCTTCTTGGCCTTGTA
Jag1a forward	CCTGTTTGGAAGGATGGGCC
Jag1a reverse	CCAGTCCACTGTACAGGGCAGAT
TP53 forward	ATGCCCCCATCATGAGCGAA
TP53 reverse	GCGCCATTGCTTTGCAAGAATTG
Notch1a forward	GCACCGGTGATGTCATCA
Notch1a reverse	GGTACCTTGCAGGTGTT
Notch1b forward	GGTGGACTTTGTCTGCAACT
Notch1b reverse	GGTGTACAGTGGCAGA
